# Arsenal of plant cell wall degrading enzymes reflects host preference among plant pathogenic fungi

**DOI:** 10.1186/1754-6834-4-4

**Published:** 2011-02-16

**Authors:** Brian C King, Katrina D Waxman, Nicholas V Nenni, Larry P Walker, Gary C Bergstrom, Donna M Gibson

**Affiliations:** 1 Department of Plant Pathology and Plant-Microbe Biology, Cornell University, Plant Science Building, Ithaca, NY 14853, USA; 2 Department of Biology, SUNY Geneseo, Geneseo, NY 14454, USA; 3 Department of Biological and Environmental Engineering, Cornell University, Riley-Robb Hall, Ithaca, NY 14853, USA; 4 USDA Agricultural Research Service, Robert W Holley Center for Agriculture and Health, Ithaca, NY 14853, USA; 5 BioWorks Inc, Victor, NY 14564, USA

## Abstract

**Background:**

The discovery and development of novel plant cell wall degrading enzymes is a key step towards more efficient depolymerization of polysaccharides to fermentable sugars for the production of liquid transportation biofuels and other bioproducts. The industrial fungus *Trichoderma reesei *is known to be highly cellulolytic and is a major industrial microbial source for commercial cellulases, xylanases and other cell wall degrading enzymes. However, enzyme-prospecting research continues to identify opportunities to enhance the activity of *T. reesei *enzyme preparations by supplementing with enzymatic diversity from other microbes. The goal of this study was to evaluate the enzymatic potential of a broad range of plant pathogenic and non-pathogenic fungi for their ability to degrade plant biomass and isolated polysaccharides.

**Results:**

Large-scale screening identified a range of hydrolytic activities among 348 unique isolates representing 156 species of plant pathogenic and non-pathogenic fungi. Hierarchical clustering was used to identify groups of species with similar hydrolytic profiles. Among moderately and highly active species, plant pathogenic species were found to be more active than non-pathogens on six of eight substrates tested, with no significant difference seen on the other two substrates. Among the pathogenic fungi, greater hydrolysis was seen when they were tested on biomass and hemicellulose derived from their host plants (commelinoid monocot or dicot). Although *T. reesei *has a hydrolytic profile that is highly active on cellulose and pretreated biomass, it was less active than some natural isolates of fungi when tested on xylans and untreated biomass.

**Conclusions:**

Several highly active isolates of plant pathogenic fungi were identified, particularly when tested on xylans and untreated biomass. There were statistically significant preferences for biomass type reflecting the monocot or dicot host preference of the pathogen tested. These highly active fungi are promising targets for identification and characterization of novel cell wall degrading enzymes for industrial applications.

## Background

The recalcitrance of lignocellulose to enzymatic degradation and the high cost of hydrolytic enzymes required for depolymerization of polysaccharides found in the plant cell wall are significant barriers to the large-scale production and commercialization of biofuels and bioproducts derived from plant biomass [1]. In order to rapidly increase production of cellulosic biofuels and bioproducts there is a need to develop more efficient and cost effective enzyme mixtures for the conversion of biomass to fermentable sugars [2]. In order to address this challenge, a better understanding of the interactions between plant cell wall polysaccharides and the diversity of cell wall degrading enzymes (CWDE) needed for efficient hydrolysis is essential.

The complexity of cell wall polysaccharides is one factor which contributes to the resistance of biomass to efficient hydrolysis for bioenergy production. Plant cell walls are heterogeneous and dynamic structures, composed of polysaccharides, proteins and aromatic polymers. Cell wall composition and structures differ among plant lineages [3]. The cell walls of Angiosperms (flowering plants) and Gymnosperms (including conifers) all contain cellulose microfibrils embedded in a matrix of pectin, hemicellulose, lignin and structural proteins, but the types and relative amounts of these structural polymers differ among groups of plants and also change as the wall matures. For example, the walls of the Poales (grasses) and other commelinoid monocots differ from dicots and non-commelinoid monocots in several ways. Type I cell walls found in non-commelinoid monocots and dicots are generally rich in xyloglucan and pectin. In contrast, the type II cell walls of commelinoid monocots contain glucuronoarabinoxylan as the major non-cellulosic polysaccharide. Commelinoid monocots are also unique in having mixed-linkage β-1,3(1,4)-glucans [3-5]. There are additional differences in the types of lignin and ferulic acid esterification found in grasses and dicots [3,6]. In addition to the physical complexity of the cell wall, it is a dynamic structure that changes as the plant grows and ages. During cell wall maturation from a primary to secondary wall in both monocots and dicots, the amounts of xyloglucans, pectins and structural proteins decrease while the amount of xylans and lignin increase. The major constituents of typical secondary cell walls are cellulose (35%-45% dry weight in grasses, 45%-50% in dicots), xylans (40%-50% in grasses, 20%-30% in dicots) and lignin (20% in grasses, 7%-10% in dicots) [4]. Thus the sugars found in cellulose and xylans are the major carbon source for fermentation of biofuels and other bioproducts.

The complexity of the plant cell wall is mirrored by the diverse arsenal of CWDE produced by lignocellulose-degrading microbes. Each type of structural polysaccharide-degrading enzyme is represented in multiple families determined by sequence and structural similarities [7-10]. The Carbohydrate-Active Enzymes Database (CAZy) categorizes cellulases (EC 3.2.1.4 and 3.2.1.91) in at least 12 different glycosyl hydrolase (GH) families, and xylanases (EC 3.2.1.8 and 3.2.1.37) in 12 GH families [11]. Some GH families contain both cellulases and xylanases (such as GH5) while others contain cellulases but no xylanases (GH7) or vice versa (GH11). Genomic analysis of lignocellulose-degrading fungi shows that a single species can have the genetic capacity to produce many different enzymes with similar functional designations (cellulase, xylanase and others). For example, the genome of the phytopathogen *Magnaporthe grisea *is predicted to encode at least 30 enzymes in six GH families for the degradation of cellulose and 44 enzymes in 11 families for the degradation of hemicellulose (Fungal Genome Initiative, Broad Institute, http://www.broadinstitute.org/science/projects/fungal-genome-initiative). When compared to six other filamentous Ascomycete fungal genomes, the industrial cellulase-producing fungus *Trichoderma reesei *has a similar number of GH families for cellulose degradation and slightly fewer GH families for hemicellulose degradation [12]. However, *T. reesei *contains the smallest total number of genes encoding cellulases and xylanases compared to six fungal genomes. The genome of the phytopathogen *Fusarium graminearum *has twice the number of genes encoding cellulases and xylanases as *T. reesei*, and the genome of *M. grisea *contains three times as many [12]. Despite having relatively few genes coding for CWDE, engineered strains of *T. reesei*, such as RUT-C30, produce large quantities of extracellular enzymes, and culture broths are highly effective at the depolymerization of cellulose because of the abundance of cellulases produced [13-15].

As a result of the complexity of lignocellulosic biomass, there is potential to supplement the limited repertoire of commercial CWDE by complementation with enzymatic diversity from other sources [12,16]. For example, the supplementation of a blend of commercial enzymes [Celluclast (from *T. reesei*) and Novozyme 188 (from *Aspergillus niger*); Novozymes A/S (Bagsvaerd, Denmark)] with culture broths from several fungal species at a level of only 10% of the total protein in the reaction was sufficient to stimulate cellulose hydrolysis to twice the benchmark activity of the commercial enzymes alone [17]. Proteins in family GH61 have recently been identified as factors that enhance the hydrolysis of lignocellulose while being weakly or non-hydrolytic by themselves [1,18]. The precise mechanism of GH61 stimulation of lignocellulose hydrolysis remains elusive, but insights from the related CBP21 that acts on insoluble chitin suggest both an oxidative and a hydrolytic step, which may result in the disruption of substrate crystallinity and increased accessibility to recalcitrant polysaccharides [19]. The genome of *T. reesei *appears to encode very few GH61 proteins compared to other filamentous Ascomycetes, including the phytopathogens *F. graminearum *and *M. grisea *[12]. Interestingly, even the genome of *Blumeria graminis *(a biotrophic phytopathogen that lacks canonical lignocellulolytic enzymes) encodes several GH61 enzymes, raising questions about the biological roles of these proteins in addition to their biotechnological applications [20].

Most plant-associated microbes (both pathogenic and saprophytic) that break down plant cell walls have the genetic capacity to produce enzymes for the degradation of the major structural polysaccharides found in the cell wall, namely cellulose, xylan and pectin [21,22]. In particular, plant pathogens have intimate relationships with their hosts, requiring penetration of the cell wall and colonization of living host tissue. Many pathogenic fungi actively kill and degrade plant tissue and utilize liberated carbohydrates for growth and reproduction. Plants produce proteins to inhibit microbial CWDE as one mechanism of disease resistance, and this interaction may drive evolution of unique enzymes in phytopathogens. For example, inhibitors of pectin-degrading enzymes are common in dicots and the pectin-rich non-commelinoid monocots [23]. These proteins have also been reported in wheat [24] and rice [25] and may be involved in control of growth and development. More commonly found in grasses are inhibitors of xylan-degrading enzymes [26-28]. Following plant senescence, pathogenic fungi may continue to colonize and overwinter on dead tissue, and many plant pathogens are also competitive saprophytes [29]. Although it is unlikely that the differences in cell wall composition between monocots and dicots are sufficient to determine host specificity, there is some evidence that plant pathogens may produce different amounts of specific CWDE depending on whether the plant host is a monocot or dicot and whether fungi are grown on cell walls from monocots or dicots [30,31].

Both plant pathogenic and non-pathogenic fungi could provide a rich source of CWDE to complement *T. reesei *and other industrial enzyme sources for biofuel and bioproduct production. In order to identify promising taxa with high hydrolytic activities for more detailed characterization and to evaluate whether the suite of CWDE produced by plant pathogens reflects host specificity, we have analysed the hydrolytic enzyme profiles of 156 species of fungi and oomycetes using multiple polysaccharide substrates. These substrates included purified cellulose and hemicellulose, pretreated biomass similar to materials for bio-refineries and untreated plant cell walls representing agricultural by-products and dedicated biofuel crops.

## Results and discussion

### Effect of carbohydrate source in growth media

An initial sampling of 12 phytopathogenic fungi was used to test the effect on CWDE production for three growth media with switchgrass (SG), soybean stem (SS) or Avicel as the primary carbon source. Data were collected for hydrolysis of nine different polysaccharide or biomass substrates. A full-factorial mixed-effect model was built with host (monocot or dicot), substrate and medium treated as fixed effects and isolate as a random effect. The third order interaction of host*substrate*medium was significant (*P *= 0.0135), as was the second order interaction of medium*substrate (*P *= 0.0184) and the primary effect of substrate (*P *= 0.0009). For all assay substrates, extracts from fungi grown on the Avicel-based medium released either comparable amounts or fewer reducing sugars than cultures grown on SG- or SS-based media, as determined by pairwise *t*-tests of fitted data from the model (see Additional File 1, Figure S1). The only example where extracts from Avicel medium were noticeably more active than the SG or SS media was the case of dicot pathogens hydrolyzing filter paper (FP). However, this difference was not significant as determined by pairwise *t*-tests (*P *= 0.0822 for dicot pathogens grown on Avicel compared to SG and *P *= 0.1097 for Avicel compared to SS).

When data from cultures grown on Avicel were removed and a mixed-effect model was fitted using standardized data from only biomass-based media (SG and SS), the second order interactions of substrate*host (*P *< 0.0001) and substrate*medium (*P *= 0.0091) were significant. The primary effects of host (*P *= 0.1319) and medium (*P *= 0.7287) were non-significant and the effect of substrate was barely non-significant (*P *= 0.0506). Extracts from cultures grown on SG medium released more sugar than cultures grown on SS medium when tested on the two xylans [arabinoxylan from oat (AXO) and xylan from birch (XY)] and the two grasses [corn stalk (CS) and SG]. The opposite trend was seen for hydrolysis of xyloglucan (XG) and the two legumes [alfalfa (AL) and SS] with cultures grown on SS-based medium releasing more sugar than cultures grown on SG-based medium. Although the model found this substrate*medium effect to be significant, pairwise *t*-tests of both standardized data and values fitted from the model were found to be non-significant. For the host*substrate interaction, extracts from dicot pathogens were significantly more active than monocot pathogens when tested for hydrolysis of the dicot substrates XG (*P *= 0.0385), SS (*P *= 0.0017) and AL (*P *= 0.0008). Other substrate*host interactions were not significant. The significant interaction between plant host and dicot substrates indicated preferential hydrolysis of monocot and dicot cell walls and host-specific cell wall polysaccharides depending on the host range of pathogens.

This preferential hydrolysis of monocot and dicot cell walls prompted us to look at host preference using a much larger data set capturing a greater diversity of fungi. Recent proteomic studies of *F. graminearum *showed that supplementation of minimal medium with pectin resulted in a total of 13 pectinases and pectate lyases being expressed, while induction with xylan induced seven pectin-degrading enzymes and of these six were induced in both cases. For xylan-degrading enzymes, supplementation with xylan induced 14 xylanases or arabinofuranosidases. Supplementation with pectin only induced seven xylan-degrading enzymes; of these, five were also induced by xylan. When the researchers supplemented minimal medium with either dicot (carrot) or monocot (maize) cell walls, the induction of pectin and xylan degrading enzymes was much more similar. When supplemented with either dicot or monocot cell walls, 12 common pectin-degrading enzymes were detected while only two additional unique enzymes were induced by dicot cell walls and only one unique pectin-degrading enzyme by maize cell walls. Similarly, both dicot and monocot cell walls induced a common set of 22 xylan-degrading enzymes, with an additional two unique enzymes induced by monocot cell walls and only a single unique enzyme induced by dicot cell walls [32]. As microbial CWDE synthesis is primarily induced by low levels of simple monosaccharides, and because these sugars are found in the majority of both monocot and dicot cell walls, it is expected that both monocot and dicot cell walls will induce similar types of enzymes by microbes [22]. This does not rule out a minor, quantitative difference in CWDE induction profiles for monocot- or dicot-based growth media, but because we observed the effect of growth medium to be subtle and not statistically significant when SS- and SG-based media were compared, cultures for the large-scale screening were grown solely on SG-based medium.

### Screening of culture collections

A total of 348 unique isolates was tested for hydrolysis of FP, three types of hemicellulose (arabinoxylan from wheat (AXW), XY and XG), biomass from two grasses (CS and SG) and biomass from two legumes (AL and SS; see Additional File 2, Table S1). Most isolates were from the kingdom Fungi (344, 98.9%). Only four (1.1%) isolates were Oomycetes in the kingdom Chromista. The majority of isolates (317, 91.1%) were in the division Ascomycota, with fewer isolates from the Basidiomycota (22, 6.3%) and the Zygomycota (5, 1.4%). At the class level, most isolates were in the Sordariomycetes (190, 54.6%) and the Dothideomycetes (91, 26.1%). The most highly represented subclasses were the Hypocreomycetidae (129, 37.1%), the Pleosporomycetidae (70, 20.1%) and the Sordariomycetidae (58, 16.7%). On the order level, the Hypocreales (121, 34.8%) and the Pleosporales (70, 20.1%) were most highly represented. The families Nectriaceae (103, 29.6%), Glomerellaceae (41, 11.8%), and Pleosporaceae (37, 10.6%) contained the greatest number of isolates. The two most sampled genera were *Fusarium *(101, 29.0%) and *Colletotrichum *(41, 11.8%).

Negative controls of extracts in the absence of substrate did not react with 3,5-dinitrosalicylic acid (DNS). Negative controls of substrates and buffer in the absence of hydrolytic enzymes detected small amounts of reducing sugar (less than 0.12 mg/mL) from the reaction of DNS with soluble compounds present in the mostly insoluble substrates, small amounts of contamination by fine particulate matter and decreased sensitivity of the DNS reaction at very low sugar concentrations. This background was subtracted from measured sugar values and negative values were adjusted to zero. The fungi tested showed a broad range of activity on eight substrates (see Additional File 3, Figure S2). *T. reesei *RUT-C30 was roughly twice as active as the top natural isolates when tested for hydrolysis of FP and SS. *T. reesei *RUT-C30 also stood out as being highly hydrolytic on the other substrates with the exception of the two xylans. Although many species were weakly or non-hydrolytic, some species exhibited activity greater or equal to *T. reesei *when assayed on either untreated biomass or xylans. Many species were represented by multiple isolates, and we observed that some isolates within a species were highly active, while others showed weaker activities. Although these studies were conducted with dried plant biomass or purified cell wall components, there have been some indications that the production of CWDE is related to fungal lifestyle, either pathogenic or saprophytic [33]. One possibility is that variability in CWDE activity might be related to isolate virulence. In a study of eight isolates of *Mycosphaerella graminicola*, several pathogenicity components were positively correlated with production of xylanase and pectinase *in vitro*, implying that CWDE may be key determinants of pathogenicity [34]. The ability of the fungus to successfully colonize plant mesophyll tissues was also strongly correlated with the production of endo-beta-1,4 xylanase activity *in planta *in a study of 26 isolates of *M. graminicola *[35].

### Hierarchical clustering

Ranking and ordering of species based on hydrolysis of diverse substrates is challenging since the activity on one substrate may be very different than on another. For example, *Sclerotinia sclerotiorum *was ranked third for hydrolysis of FP but 39th for hydrolysis of XY. Hierarchical clustering was used to provide a useful way to organize the moderately large dataset into meaningful groups and to identify coherent patterns. Clustering of the complete dataset (excluding *T. reesei*) identified two major groups of species (see Additional File 4, Figure S3). The top tier of 86 (55%) species showed moderate or strong hydrolysis of most substrates tested. A bottom tier contained 69 (45%) inactive or very weakly active species. All species tested from the genera *Bipolaris *(4), *Colletotrichum *(7), *Penicillium *(3), *Rhizoctonia *(4), *Sclerotinia *(4) and *Trichoderma *(5) were in the top tier of active isolates. The genus *Fusarium *was represented by 20 species, the greatest number of any genus. Most species of *Fusarium *(17) fell in the top tier of active species. However, three species (*F. decemcellulare*, *F. lateritium *and *F. merismoides*) were average or below average and fell in the bottom tier. Of the nine species tested within the genus *Aspergillus*, four species (*A. lineolatus*, *A. awamori*, *A. fumigatus *and *A. niger*) clustered in the active group, while five species (*A. candidus*, *A. janus*, *A. penicilloides*, *A. peyronelii *and *A. proliferans*) fell in the bottom tier.

**Figure 1:**
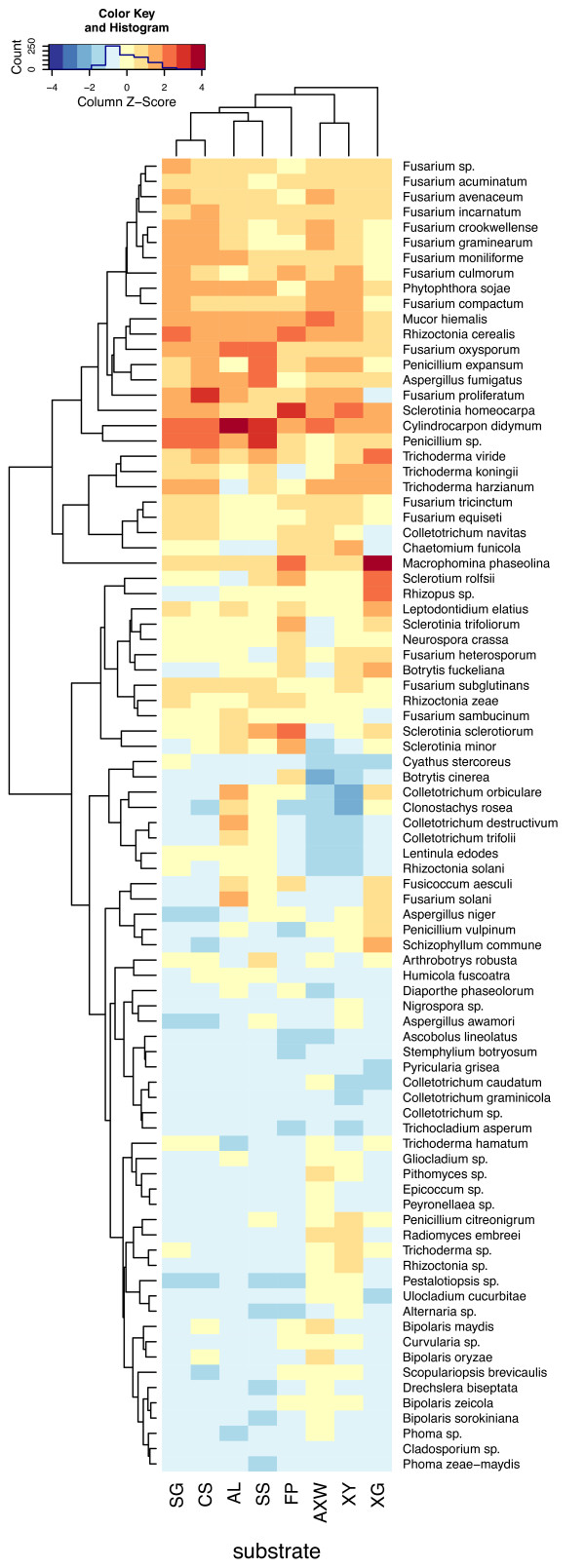
**Hierarchical clustering of active species**. Heatmap showing the mean activities and clustering of 86 species of plant pathogenic and non-pathogenic fungi when assayed for hydrolysis of eight polysaccharide substrates [XG, xyloglucan (from tamarind); FP, filter paper; AL, alfalfa; SS, soybean stems; SG, switchgrass; CS, corn stalks; AXW, arabinoxylan (from wheat); XY, xylan (from birch)]. Weakly and inactive species were excluded. *Trichoderma reesei *RUT-C30 was excluded because of its unusual hydrolytic activities. Negative estimations of reducing sugars were adjusted to zero and data were standardized within substrates by subtracting the substrate mean and dividing by the standard deviation. Dendrogram and ordering was determined using the distance matrix computation (dist) and hierarchical clustering (hclust) functions in R. Red colors indicate values greater than the substrate mean, while blue colors indicate values less than the mean. Column Z-score and color intensity indicate how many standard deviations the species mean is from the substrate mean.

Data from the weakly active tier of species was excluded, and the top tier containing 86 species with moderate or strong hydrolytic activities was used for further analysis, revealing a cluster of 27 very highly active species (Figure 1). In particular, the species *F. proliferatum*, *F. oxysporum*, *A. fumigatus*, *Penicillium expansum*, *Mucor hiemalis*, *Rhizoctonia cerealis*, *S. homeocarpa*, *Cylindrocarpon didymum*, *T. viride*, *Macrophoma phaseolina *and *Penicillium *sp. had activities greater than two standard deviations from the mean for at least one of the eight substrates tested and also had high activity on most other substrates. In addition to these broadly active species, *Sclerotium rolfsii *and *Rhizopus *sp. had activity greater than two standard deviations from the mean for hydrolysis of XG and *S. sclerotiorum *had activity greater than two standard deviations from the mean for hydrolysis of FP. However, these three isolates were not extremely active across a broad range of substrates. Several species of *Fusarium *(*Fusarium *sp., *F. acuminatum*, *F. avenaceum*, *F. incarnatum*, *F. graminearum*, *F. crookwellense*, *F. moniliforme*, *F. culmorum*, *F. compactum*, *F. proliferatum *and *F. oxysporum*) clustered together and were highly active on grass cell walls and xylans, as well as being higher than average on most other substrates. Three species of *Trichoderma *(*T. viride*, *T. koningii*, and *T. harzianum*) clustered together and had very high activity on XG and good activity on other substrates. Some species, such as all four species of *Bipolaris*, had activities near or above the mean for AXW and XY, but were average or below average for other substrates. All isolates showed growth on quarter strength potato dextrose agar (PDA), but it is possible that the use of a minimal medium, based on SG where biomass is the major carbon source, may have been sub-optimal for growth of some fungi. Negative results from this study do not rule out a species as producing active CWDE. Nevertheless, these data identify numerous species that are very capable of hydrolyzing cellulose, hemicellulose and lignocellulosic biomass.

In addition to identifying clusters of species with similar activities, the substrates also clustered showing biological significance. The two grass substrates (CS and SG) clustered together as did the two legume substrates (AL and SS). These two groups of cell walls [grass (monocot) and legume (dicot)] are clearly distinct from each other as reflected in hydrolysis by diverse microbes. This also indicates that some suites of enzymes produced by microbes are more suited for breaking down grass cell walls, while other suites of enzymes are more suited for breaking down legume cell walls. Both xylans tested (XY and AXW) clustered together. FP and XG did not cluster closely with any other substrates.

**Figure 2:**
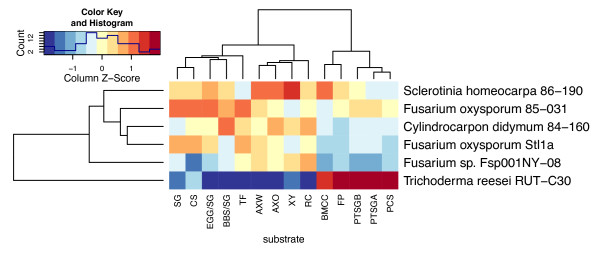
**Hierarchical clustering of top isolates and *Trichoderma reesei***. Heatmap showing mean activities and clustering of the top isolates on each substrate and *T. reesei *when assayed for hydrolysis of 14 polysaccharide substrates [SG, switchgrass; CS, corn stalk; EGG/SG, eastern gammagrass/switchgrass mix; BBS/SG, big bluestem/SG mix; TF, tall fescue; AXW, arabinoxylan (wheat); AXO, arabinoxylan (oat), XY, xylan (birch); RC, reed canary grass; BMCC, bacterial microcrystalline cellulose; FP, filter paper; PTSGB, base pretreated SG; PTSGA, acid pretreated SG; PCS, acid pretreated corn stover]. Each substrate was hydrolyzed for two lengths of time and the mean of those two timepoints was used for clustering. Selected isolates showed the highest activity on at least one substrate and at least one timepoint. Measured reducing sugars were standardized within substrates by subtracting the substrate mean and dividing by the standard deviation. Dendrogram and ordering was determined using the distance matrix computation (dist) and hierarchical clustering (hclust) functions in R. Red colors indicate values greater than the substrate mean, while blue colors indicate values less than the mean. Column Z-score and color intensity indicate how many standard deviations the isolate mean is from the substrate mean.

A smaller set of top isolates was recultured and tested using a greater number of substrates and hydrolysis times. Data from a subset of isolates with the highest activity for each substrate and time were standardized within each substrate and time. The standardized values were averaged for the two timepoints, and organized using hierarchical clustering (Figure 2). Among the top plant pathogenic isolates, *S. homeocarpa *86-190 showed very good activity on isolated cellulose and xylans, as well as most types of grass cell wall biomass. *F. oxysporum *85-031 was above average on both untreated and pretreated grasses. The hypercellulolytic mutant, *T. reesei *RUT-C30, shows an unusual pattern of hydrolytic activity. It was highly active on the two pure cellulosic substrates tested [FP and bacterial microcrystalline cellulose (BMCC)] as well as the three pretreated biomass samples [acid pretreated corn stover (PCS), acid pretreated switchgrass (PTSGA) and base pretreated switchgrass (PTSGB)]. However, when compared with the other top isolates, *T. reesei *was the weakest isolate for hydrolysis of the three xylans (AXW, AXO, XY) and five of six types of untreated grass cell walls [SG, eastern gammagrass/switchgrass mix (EGG/SG), big bluestem/switchgrass mix (BBS/SG), tall fescue (TF) and reed canarygrass (RC)]. This clearly illustrates the skewed hydrolytic profile of *T. reesei*, which emphasizes cellulase production that is essential for hydrolysis of pretreated grasses where cellulose is the major component. However, high production of cellulases may result in relatively ineffective hydrolysis of the more complex and heterogeneous untreated plant cell walls in which lignin and hemicellulose may limit cellulose accessibility. For hydrolysis of untreated biomass, high xylanase activity could directly increase the amount of five carbon sugars (mainly xylose and arabinose) as well as stimulate cellulose hydrolysis, perhaps by improving cellulose accessibility. Increased hydrolysis of glucans in pretreated grass cell walls has been reported by supplementing *T. reesei *cellulases with endoxylanase, arabinofuranosidase, α-glucuronidase, acetyl xylan esterase, ferulic acid esterase and other activities [36-38]. Accessory enzymes that facilitate more complete utilization of plant biomass could be used to develop less energetically and chemically intensive pretreatments and allow for greater fermentable sugar recovery, especially for five carbon sugars derived from hemicellulose.

### Mixed-effect modelling for lifestyle and host specificity

Species identified as being weakly active when tested on most substrates in the initial clustering of all 155 species were excluded from further statistical testing (see Additional File 4, Figure S3). As some taxa were sampled more frequently than others (for example, *Fusarium*), a mixed-effect model was used to compare groups of fungi for lifestyle (pathogenic/non-pathogenic) and host specificity (monocot/dicot) where genus, species and isolate are treated as random effects. By treating taxonomic ranks as random, increased sampling within a taxonomic group will not affect the mean of the larger grouping (lifestyle and host specificity) but will give a more accurate estimation of variance for the group. In the top tier of 86 active species, 17 species could confidently be identified as pathogens primarily of dicots, 28 as pathogens of monocots and 16 as non-pathogenic (Table 1). Among these, several genera (*Colletotrichum*, *Fusarium*, *Phoma *and *Sclerotinia*) included some species pathogenic on monocots and others pathogenic on dicots. The large number of pathogenic species with known host specificity and the number of species classified as non-pathogenic provides a robust data set to use mixed-effect modelling to test whether hydrolysis of plant cell walls and cell wall polysaccharides reflects a difference between pathogenic and non-pathogenic lifestyles and for pathogenic species if hydrolysis reflects host preference.

**Table 1:** Species used for comparison of lifestyle (pathogenic or non-pathogenic) and host specificity (monocot or dicot).

Dicot	Monocot	Non-pathogenic
*Botrytis cinerea*	*Bipolaris maydis*	*Aspergillus fumigatus*
*B. fuckeliana*	*B. oryzae*	*A. niger*
*Colletotrichum destructivum*	*B.sorokiniana*	*Clonostachys rosea*
*C. orbiculare*	*B. zeicola*	*Epicoccum sp.*
*C. trifolii*	*Colletotrichum caudatum*	*Humicola fuscoatra*
*Diaporthe phaseolorum*	*C. graminicola*	*Lentinula edodes*
*Fusarium incarnatum*	*C. navitas*	*Mucor hiemalis*
*F. oxysporum*	*C. sp.*	*Neurospora crassa*
*F. sambucinum*	*Cyathus stercoreus*	*Nigrospora sp.*
*F. solani*	*Drechslera biseptata*	*Penicillium vulpinum*
*Phoma sp.*	*Fusarium acuminatum*	*Trichocladium asperum*
*Phytophthora sojae*	*F. avenaceum*	*T. hamatum*
*Sclerotinia minor*	*F. crookwellense*	*T. harzianum*
*S. sclerotiorum*	*F. culmorum*	*T. koningii*
*S. trifoliorum*	*F. equiseti*	*T. sp.*
*Stemphylium botryosum*	*F. graminearum*	*T. viride*
*Ulocladium cucurbitae*	*F. heterosporum*	
	*F. moniliforme*	
	*F. proliferatum*	
	*Fusarium sp.*	
	*F. subglutinans*	
	*F. tricinctum*	
	*Phoma sp.*	
	*Phoma zeae-maydis*	
	*Pyricularia grisea*	
	*Rhizoctonia cerealis*	
	*R. zeae*	
	*Sclerotinia homeocarpa*	

All species were in the top tier of 86 active isolates as determined by clustering analysis using the complete data set. This subset of species was used to test the effect of lifestyle (pathogenic/non-pathogenic) and host specificity (monocot/dicot) on hydrolysis of eight polysaccharides and plant cell walls. For testing lifestyle, three additional pathogens of woody species were also included, *Cylindrocarpon didymum*, *Fusicoccum aesculi *and *Schizophyllum commune*.

**Figure 3:**
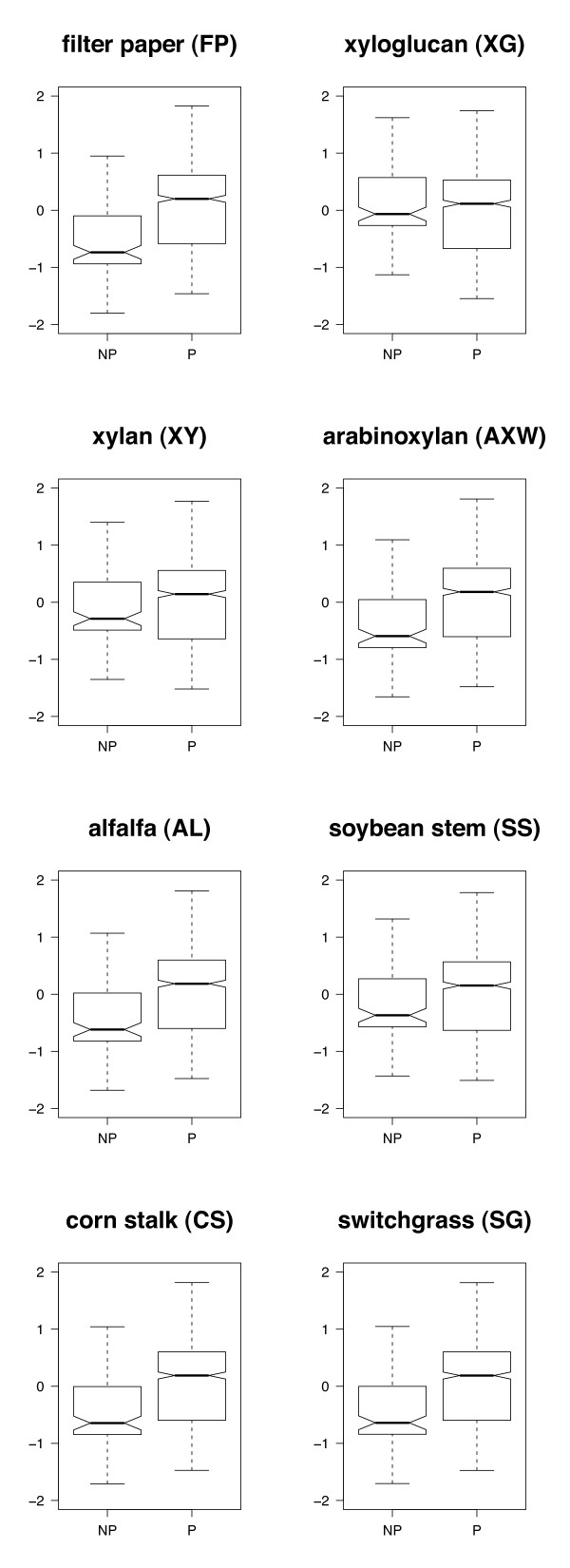
**Interactions between lifestyle (pathogenic or non-pathogenic) and substrates**. Fitted values from mixed-effect model on activity standardized within substrates. Lifestyle [non-pathogenic (NP) or pathogenic (P)], substrate (FP, XG, XY, AXW, AL, SS, CS, SG) and the lifestyle*substrate interaction were treated as fixed effects; genus/species/isolate were nested random effects. Data used for this analysis included 47 species of pathogens and 16 species of non-pathogens. Non-pathogenic and pathogenic species are the same as presented in Table 1 with the addition of three pathogens of woody species, *Cylindrocarpon didymum*, *Fusicoccum aesculi *and *Schizophyllum commune*. Pathogenic species had significantly higher activity on the substrates FP, AXW, AL, SS, CS and SG as determined by pairwise *t*-tests on fitted values from the model (*P *< 0.005). There was no significant difference on XG (*P *= 0.059) and XY (*P *= 0.08). The middle black bar at the center of the notch indicates the median value, edges of boxes indicate the interquartile range and whiskers indicate minimum and maximum values.

A mixed-effect model was fitted using lifestyle (pathogen or non-pathogen), substrate (FP, XG, XY, AXW, AL, SS, CS, SG) and the lifestyle*substrate interaction as fixed effects. Genus, species and isolate were treated as nested random effects. Lifestyle by itself was not significant (*P *= 0.5346), but substrate (*P *< 0.0001) and the lifestyle*substrate interaction (*P *< 0.0001) were highly significant, indicating that on at least one substrate there was a significant difference between activities of pathogens and non-pathogens. Pairwise *t*-tests comparing pathogens and non-pathogens for each substrate found that pathogens were more hydrolytic on six of eight substrates (*P *< 0.005, Figure 3). There was no significant difference between pathogens and non-pathogens when tested on XG (*P *= 0.059) or XY (*P *= 0.08). Although more isolates of pathogenic fungi than non-pathogens were tested, treating genus, species and isolate as random effects reduced the sampling bias; these results indicate a significant trend and highlight the powerful suite of CWDE produced by many plant pathogenic fungi. Not only do pathogens often rely on CWDE for breaching the physical barrier presented by plant cell walls and rapid colonization of plant tissue, many pathogens are also capable of saprophytic growth on senesced plant tissue. Efficient CWDE may allow plant pathogens to quickly colonize dead plant material and outcompete environmental saprophytes and also provide a carbon source required for growth and reproduction.

**Figure 4:**
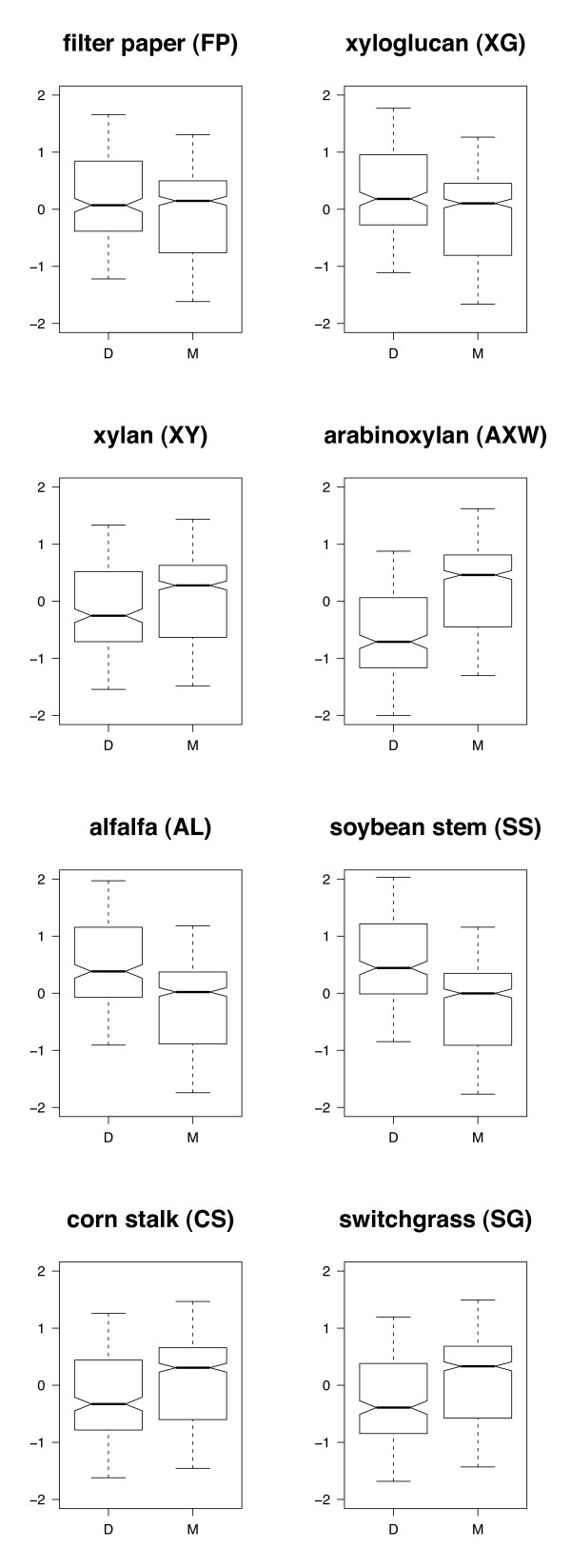
**Interactions between hosts (monocot or dicot) and substrates**. Fitted values from mixed-effect model on activity standardized within substrates. Host preference of pathogenic fungi [dicot (D) or monocot (M)], substrate (FP, XG, XY, AXW, AL, SS, CS, SG) and the host*substrate interaction were treated as fixed effects; genus/species/isolate were nested random effects. Data used for this analysis included 17 species of dicot pathogens and 28 species of monocot pathogens. Dicot pathogens had significantly higher activity on the substrates FP, XG, AL and SS as determined by pairwise *t*-tests on fitted values from the model (*P *< 0.0005). Monocot pathogens had significantly higher activity on the substrates XY, AXW, CS and SG (*P *< 0.001). The middle black bar at the center of the notch indicates the median value, edges of boxes indicate the interquartile range and whiskers indicate minimum and maximum values.

A similar model to the pathogenic/non-pathogenic lifestyle model was fitted using host preference (monocot or dicot) in place of lifestyle. Both fixed effects of host (*P *= 0.0071) and substrate (*P *< 0.0001) were significant, as was the host*substrate interaction (*P *< 0.0001). When pairwise *t*-tests were performed comparing pathogens of monocots and dicots on each substrate, there were significant differences in all cases (*P *< 0.001, Figure 4). Pathogens of dicots showed slightly greater hydrolysis of FP, and although the difference was significant, the median value of monocot pathogens was greater than the median value for dicot pathogens. Similarly, monocot pathogens showed significantly greater hydrolysis of XY than dicot pathogens, but the interquartile ranges for the two host groups were nearly the same. However, for dicot cell walls (AL and SS) and dicot-specific hemicellulose (XG), pathogens of dicots clearly showed greater hydrolytic activity than pathogens of monocots. In contrast, for monocot cell walls (CS and SG) and monocot-specific hemicellulose (AXW), pathogens of monocots clearly showed greater hydrolytic activity than pathogens of dicots. These results provide strong evidence that among plant pathogens with the capacity to degrade plant cell walls, pathogens of monocots are better adapted for degradation of monocot cell walls while pathogens of dicots are better adapted for degradation of dicot cell walls, reflecting host preferences.

The observation that pathogens of monocots are better adapted for degradation of monocot cell walls may have industrial applications for the processing of mixed biomass containing a variety of plant types, including grasses and legumes. By tailoring industrial enzyme mixtures similarly to the way in which plant pathogens have evolved specialized CWDE systems for monocots and dicots, it may be possible to achieve more efficient hydrolysis of diverse biomass feedstocks. Although most lignocellulolytic fungi have the capacity to degrade most plant cell wall polysaccharides, these results provide compelling evidence for the adaptation of phytopathogens for degradation of cell walls and hemicellulose from their host of preference. A few genera were well represented with multiple species and isolates, allowing a comparison among species within a genus with multiple hosts. The genera *Colletotrichum *and *Fusarium *contain some species pathogenic on monocots and other species pathogenic on dicots. In the genus *Bipolaris*, all species tested were pathogens of monocots. When hydrolytic preferences were examined at the species level within these genera, similar results were observed when all data were considered suggesting that host specificity is reflected in hydrolytic activity at the species level within a genus.

One challenge when screening an unknown pool of organisms for biological activity is capturing the greatest variation in a reasonable number of samples. In order to assess the contribution of taxonomic rank on variance in the data, a mixed-effect model was fitted for the response of standardized activity with family as a fixed effect. Genus, species and isolate were treated as nested random effects. *T. reesei *and the bottom tier of 69 weakly active species were excluded from the dataset. Based on hydrolysis of eight substrates by 248 isolates of plant pathogenic and non-pathogenic fungi from 86 species and 45 genera, taxonomic hierarchy (genus, species, isolate) accounted for roughly 42% of the total observed variance. Genus-to-genus variance contributed 5.3% to the total variance, species-to-species variance accounted for 17.8% of the total variance and isolate-to-isolate variance accounted for 18.5% of the total variance. This indicates that greater variation is seen at the species and isolate level and further screening at the sub-generic level will probably reveal significant variation. Therefore, after the identification of promising genera, further screening within the genus will probably detect a wide range in activity. Similarly, after the identification of promising species, there is still significant variation among isolates which may reveal superior candidates. This approach is warranted, particularly if a genus or species is suspected of being hyper-variable. Such variability is commonly seen when phytopathogenic isolates are examined for virulence traits [39,40]. In *Cochliobolus carbonum*, a targeted gene knockout of a regulatory gene resulted in mutants with low levels of pectinases and other CWDE and with reduced virulence on maize, supporting that these enzymes play an important role in pathogenicity [41]. Virulence may be a useful selection criterion with which to identify the most promising isolates as is suggested by the relationship between the positive correlation of CWDE and measures of pathogenicity, such as lesion size and disease development [33-35]. This study itself does not directly explore sources of variance, however it could be due to many factors including multiple isoforms or copies of similar enzymes, variation in total production or secretion of hydrolytic enzymes and different suites of CWDE diversity. Earlier work to identify virulence factors in plant pathogens pointed to the presence of multiple forms of enzymes with similar functions, such as pectinases [33,42], that were associated with infection of living plant tissues. Recent surveys of fungal genomes clearly indicate that these organisms are replete with many types of CWDE classes and genes [12]. Most pathogenic isolates tested were isolated directly from the field, and many of these isolates had comparable or higher hydrolytic activity than the engineered strain *T. reesei *RUT-C30 when tested on untreated biomass and hemicellulose. This natural diversity of CWDE could provide a large reservoir that can be further improved by engineering enzymes and strains for increased performance.

## Conclusions

The results presented here clearly illustrate that plant pathogens are promising sources in which to discover highly active CWDE that would be useful for more efficient lignocellulosic digestion. Genomic analysis of several plant pathogens indicates an abundance of CWDE, particularly when compared with *T. reesei *[12]. While *T. reesei *produces a CWDE system that results in efficient hydrolysis of pure cellulose and pretreated biomass, this high cellulase activity does not confer exceptional hydrolysis of untreated plant biomass. Several plant pathogens were identified as highly competent degraders of untreated biomass. Compared to *T. reesei*, many plant pathogens had higher xylanase activity and some highly active isolates had greater activity than *T. reesei *when tested on grass cell walls. Specifically, the species *F. avenaceum*, *F. incarnatum*, *F. graminearum*, *F. crookwellense*, *F. moniliforme*, *F. culmorum*, *F. compactum*, *F. proliferatum*, *F. oxysporum*, *Phytophthora sojae*, *A. fumigatus*, *P. expansum*, *M. hiemalis*, *R. cerealis*, *S. homeocarpa*, *S. sclerotiorum*, *S. trifoliorum*, *C. didymum*, *T. viride*, *T. koningii*, *T. harzianum*, *Chaetomium funicola*, *M. phaseolina*, *S. rolfsii*, *Leptodontium elatius*, as well as the unidentified species *Fusarium *sp., *Penicillium *sp. and *Rhizopus *sp. are promising candidates in which to discover highly active enzymes in one or more classes of CWDE. Although we did not test synergism directly, enhancement of *T. reesei *cellulases with crude enzyme preparations from other fungi has been documented and may lead to the identification of novel accessory enzymes for biomass hydrolysis [1,17,18,43]. Any of the top candidates identified in this study would be good candidates for closely controlled synergy experiments in future work. Some of these taxa may contain novel enzymes with unique activity, such as GH61. In addition, a closer examination of the CWDE systems employed by these naturally highly active taxa may provide insights to guide the engineering of multi-enzyme cocktails based on established enzymatic activities and synergies.

Pathogenic species showed greater hydrolysis than non-pathogenic species for all substrates tested except xyloglucan from tamarind and xylan from birch, on which there was no significant difference between the groups. Among pathogenic species, pathogens of monocots had relatively higher hydrolysis of monocot hemicellulose (arabinoxylan) and cell walls (corn stalk and switchgrass), while pathogens of dicots preferentially hydrolyzed dicot hemicellulose (xyloglucan) and cell walls (alfalfa and soybean stem). Together, these results show that many plant pathogenic fungi are highly competent producers of lignocellulolytic enzymes, specialized on their preferred hosts, and a promising source from which to find accessory enzymes that may complement the highly cellulolytic CWDE system of *T. reesei*.

## Methods

### Cultures and growing conditions

A total of 348 isolates from 156 species in 93 genera were used in this study (see Additional File 2, Table S1). Unless noted otherwise, isolates were obtained from the New York Field Crop Pathogen Culture Collection (NYFC, Gary Bergstrom, Cornell University, Ithaca, NY, USA) or the Cornell Plant Pathology Teaching Culture Collection (CPP, David Kalb, Cornell University, Ithaca, NY, USA). Frozen stocks of spores and/or mycelium were stored in 20% glycerol at -80°C. Isolates were plated on quarter strength potato dextrose agar (PDA; 6 g potato dextrose broth (Beckton Dickinson, Franklin Lakes, NJ, USA), 16 g agarose, 1L H_2_O) and grown for seven days at 25°C. Five subcultures of each isolate were made by transferring 6 mm plugs from PDA cultures to biomass- or cellulose-based agar media modified from the ATCC cellulose medium 907 (0.5 g (NH_4_)_2_SO_4_, 0.5 g L-asparagine, 1 g KH_2_PO_4_, 0.5 g KCl, 0.2 g MgSO_4_, 0.1 g CaCl_2_, 0.5 g yeast extract, 16 g agarose, 5 g cellulose or biomass, 1 L H_2_O) in 50 mm Petri dishes. For use as the carbon source in the cellulose-based agar medium, Avicel (FMC BioPolymer, type PH-101, 50 mm, Philadelphia, PA, USA) was used. For biomass-based media, dry switchgrass (SG; *Panicum virgatum *cv. 'Blackwell', 15 + 4 year stands, Pawling, NY, USA) and soybean stems (SS; *Glycine max *from Phil Atkins, Cornell University Department of Crop and Soil Sciences, Ithaca, NY, USA) were milled to pass through a 20 mesh screen. Cultures were grown on these media for an additional 10 days before freezing at -80°C. Cultures were then thawed and chopped into roughly 1 cm^2 ^pieces and extracted in 11 mL of buffer (0.1 M Na acetate, 0.02 M NaCl, 0.02% Na azide, pH 5.5) for 2 h at room temperature: 1.5 mL aliquots of extracts were placed into individual wells of 2 mL 96-deepwell plates (Eppendorf AG, Deepwell Plate 96, 2000 μL, Hamburg, Germany) and frozen at -80°C until assayed.

### Enzyme assays

Hydrolysis of various polysaccharide substrates was conducted in 96 well microplates [44]. Two cellulosic substrates were used: FP (7 mm discs of Whatman No. 1 1001070, Maidstone, UK) and BMCC (CPKelco Cellulon Press Cake, K5C486-SC3 010133A, Atlanta, GA, USA). Four hemicellulosic substrates were used: XY (Sigma X0502, St. Louis, MO, USA), AXO (Sigma X0627, MO, USA), AXW (Megazyme P-WAXYI, Wicklow, Ireland) and XG (Megazyme P-XYGLN, Wicklow, Ireland). Several types of untreated biomass milled to 0.5 mm were tested: CS (*Zeae mays *from Gary Bergstrom, Cornell University Department of Plant Pathology and Plant-Microbe Biology, Ithaca, NY, USA), SG (see previous description), SS (see previous description), as well as AL (*Medicago sativa *cv. 'Oneida VR'), RC (*Phalaris arundinacea *cv. 'Bellevue'), TF (*Festuca arundinacea *cv. 'Bull'), biomass from a mixed BBS/SG stand (*Andropogon gerardii *cv. 'Bonanza'/*P. virgatum *cv. 'Cave-in-Rock'), and from a mixed EGG/SG stand (*Tripsacum dactyloides *cv. 'Pete'/*P. virgatum *cv. 'Cave-in-Rock'), all of which were provided by Hilary Mayton (Cornell University Department of Plant Breeding and Genetics, Ithaca, NY, USA) unless otherwise noted. Three types of pretreated biomass were tested: dilute base pretreated SG (PTSGB; same batch of SG as previously described), dilute acid pretreated SG (PTSGA; same batch of SG as previously described) and dilute acid pretreated CS (PCS; from Daniel Schell, National Renewable Energy Laboratory, CO, USA). SG pretreatment was performed in 10 g batches. For dilute base pretreatment, a 5% SG (w/w) suspension in 1% NaOH (w/w) was incubated at 25°C and shaken at 200 rpm for 24 h. For dilute acid pretreatment of SG, a 7% SG suspension in 0.75% H_2_SO_4 _was autoclaved at 121°C for 1 h. Pretreatments were neutralized afterwards. All biomass samples and BMCC were washed at least five times by centrifugation in three volumes of distilled water to remove background simple sugars prior to use in assays.

Hydrolysis reactions were conducted by mixing fungal extracts in a 1:1 (v/v) ratio with 2% substrate (dry weight substrate/total volume of suspension or solution). For insoluble substrates, the total reaction volume was 180 μL and was conducted in flat-bottom microplates (Corning Life Sciences, Costar flat bottom 3370, Corning, NY, USA). Insoluble slurries were prepared in small beakers and kept under constant agitation with magnetic stir bars while pipetted by hand into microplates using truncated pipette tips (Laboratory Product Sales Inc, L111806, Rochester, NY, USA). For soluble substrates, the total reaction volume was 50 μL and was conducted in conical-bottom microplates (Eppendorf AG, 96-well twin.tec, Hamburg, Germany). Plates were sealed with aluminum sealing film (Axygen, PCR-AS-200, Union City, CA, USA) and incubated at 37°C for various times depending on the substrate and experiment, then frozen. In order to test the effect of growth medium, nine substrates were used (FP, CMC, XY, AXO, XG, SG, CS, SS, AL). Hydrolysis of AXO and XY was conducted for 1 h, XG for 2 h, CMC for 6 h, FP for 24 h and SG, CS, SS and AL for 72 h. The major screening of all 348 isolates was conducted by culturing five replicates of each isolate on SG agar. Extracts were tested for hydrolysis of eight substrates (AL, AXW, CS, FP, SG, SS, XG, XY). XY was hydrolyzed for 1 h, XG for 2 h, AXW for 10 h, FP for 24 h and SG, CS, SS and AL for 72 h. Fresh extracts from the top isolates were re-cultured and re-tested. Extracts from these cultures were tested on a broad range of substrates (AXW, AXO, XY, FP, BMCC, SG, CS, RC, TF, BBS, EGG, SGA, SGB, PCS). For each substrate, hydrolysis was stopped at two time points. AXO and XY were hydrolyzed for 1 h and 3 h, AXW for 3 h and 11 h, BMCC for 18 h and 72 h, and all other substrates for 72 h and 168 h.

### Carbohydrate analysis

The colorimetric assay based on 3,5-dinitrosalicylic acid (DNS) for estimating total reducing sugars was employed in a microplate format [44-47]. The DNS reagent was prepared by dissolving 10.6 g of DNS and 19.8 g NaOH in 1416 mL ddH_2_O, then adding 306 g Rochelle salts (NaK tartrate), 7.6 mL phenol, and 8.3 g Na metabisulphite [48]. DNS reagent was allowed to sit for one week prior to use. Following completion of enzymatic reactions, 50 μL of hydrolysate was carefully removed in order to avoid disturbing and pipetting any insoluble undigested substrate, and added to 100 μL of DNS solution in 96 conical-well plates (Eppendorf AG, 96-well twin.tec, Hamburg, Germany). For BMCC, plates were centrifuged to compress the non-hydrolyzed substrate prior to removal of hydrolysate. In order to minimize plate-to-plate variation, each plate contained three replicates of sugar standards in buffer for the linear range of the DNS assay (0, 0.5, 1.0, 1.5, 2.0 and 3.0 mg/mL glucose or 0, 0.5, 1.0, 1.5, 2.0 and 2.5 mg/mL xylose). Plates were sealed with silicone compression mats (Axygen, CM-FLAT, Union City, CA, USA) and heated in a thermocycler (MJResearch Inc, PTC-100, Waltham, MA, USA) for 5 min at 95°C followed by cooling and holding at 20°C. If the initial analysis of hydrolysate contained more sugar than the linear range of the DNS assay, samples were retested by a double dilution before adding DNS reagent. For analysis, 36 μL of the completed DNS reaction were diluted in 160 μL ddH_2_O in a flat-bottom microplate. Absorbencies were measured at 540 nm and sugar concentrations were calculated from a linear regression of the standards. Pipetting was either done manually using a multichannel pipettor, or was automated using an epMotion 5075 liquid handling system (Eppendorf, Hamburg, Germany).

### Statistical analysis

Data were analysed using the software package R (R Development Core Team, Vienna, Austria, 2009). An initial group of 12 monocot pathogens and 12 dicot pathogens were arbitrarily selected from the NYFC culture collection to represent a diversity of fungal species. Each isolate was replicated three times on each of the three media based on Avicel, SG and SS. Extracts were assayed for hydrolysis of nine substrates (AL, AXO, CMC, CS, FP, SG, SS, XG, XY). Of the initial 24 isolates selected, the 12 showing the greatest hydrolysis were selected for further analysis. These included six pathogens of grasses and six pathogens of legumes (see Additional File 2, Table S1).

Raw data were corrected by subtracting background reducing sugars from each substrate as determined by an enzyme-free buffer blank. In order to facilitate direct comparisons among substrates, sugar concentrations were standardized within substrates by centering and scaling the data (subtracting the mean and dividing by the standard deviation). A linear mixed-effects model was fit for the response standardized activity starting with a full factorial model using the effects host (monocot or dicot), medium (Avicel, SG, SS), and substrate (FP, CMC, XY, AXO, XG, AL, SS, CS, SG) as fixed effects and isolate as a random effect. The analysis was repeated excluding data from Avicel-grown cultures.

For the large-scale screening of 348 unique isolates, five cultures were extracted for each isolate. Extracts were assayed for hydrolysis of eight substrates (AL, AXW, CS, FP, SG, SS, XG, XY). The mean background sugars were subtracted from the data for each substrate and data were again centered and scaled. Data from all isolates except *T. reesei *RUT-C30 were ordered and grouped using the distance matrix computation (dist) and hierarchical clustering (hclust) functions in R. Heatmaps were generated using the function heatmap.2 in the gplots package. The package RColorBrewer was used for colorizing the heatmaps. The hypercellulolytic mutant *T. reesei *RUT-C30 was excluded from this analysis because of its unusual hydrolytic activity on cellulosic materials compared to natural isolates.

In order to test the interaction of lifestyle (pathogen or non-pathogen) and host preference (monocot or dicot) with substrates, a subset of isolates was created for species in the top tier identified by clustering analysis and those which could be confidently assigned a host (monocot, dicot or non-pathogen). All pathogens of monocots were pathogens of commelinoid monocots with the exception of *F. oxysporum *f. sp. *tulipae *that is a pathogen of tulip, a non-commelinoid monocot with cell walls resembling dicots. This isolate was treated as a dicot pathogen for statistical analysis. Data were standardized by centering and scaling. A mixed-effect model was built for the response of standardized activity using the fixed effects of lifestyle or host, substrate and the interaction lifestyle*substrate or host*substrate. Genus, species and isolate were treated as nested random effects. The linear mixed-effects model (lme) function in the package nlme was used for all modelling.

A mixed-effect model was fitted in order to test the effect of taxonomic hierarchy as a source of variation in hydrolytic activity. Data from all isolates in the top tier (excluding *T. reesei *RUT-C30) were standardized by centering and scaling. Family was treated as a fixed effect, while genus, species and isolate were treated as nested random effects. Variance components were calculated as a percentage of total variance by squaring the standard deviations of each random effect, and dividing by the total variance.

## Abbreviations

AL: alfalfa; AXO: arabinoxylan from oat; AXW: arabinoxylan from wheat; BBS/SG: big bluestem/switchgrass mix; BMCC: bacterial microcrystalline cellulose; CAZy: carbohydrate active enzyme database; CS: corn stalk; CWDE: cell wall degrading enzyme(s); DNS: 3,5- dinitrosalicylic acid; EGG/SG: eastern gammagrass/switchgrass mix; FP: filter paper; GH: glycosyl hydrolase; PCS: acid pretreated corn stover; PTSGA: acid pretreated switchgrass; PTSGB: base pretreated switchgrass; RC: reed canarygrass; SG: switchgrass; SS: soybean stem; TF: tall fescue; XG: xyloglucan from tamarind; XY: xylan from birch.

## Competing interests

The authors declare that they have no competing interests.

## Authors' contributions

KDW, NVN and BCK cultured the organisms for enzyme extraction and KDW isolated some of the organisms used in the study. BCK and NN performed the screening assays. BCK carried out the statistical analysis and drafted the manuscript. LPW, GCB and DMG conceived the study and participated in its design and coordination and helped to draft the manuscript. All authors read and approved the final manuscript.

## Supplementary Material

Additional file 1 **Supplemental Figure 1**. Interactions among hosts, growth media, and substrates. Fitted values from mixed-effect model on activity standardized within substrates. Data are from six dicot (d) pathogens and six monocot (m) pathogens grown on Avicel (A), switchgrass (SG) and soybean-stem (SS) supplemented minimal media. The lower case letter on the *x*-axis label indicates pathogen host (d, m) and the upper case letters indicate growth media (A, SG, SS). Each set of plots is for nine different substrates (FP, CMC, XY, AXW, XG, AL, SS, CS, SG). The effects of host, media and substrate, as well as their interactions, were treated as fixed effects and isolate was treated as a random effect. The third order interaction of host*substrate*medium was significant (*P *= 0.0135), as was the second order interaction of medium*substrate (*P *= 0.0184) and the primary effect of substrate (*P *= 0.0009). For all assay substrates, extracts from fungi grown on the Avicel-based medium released either comparable amounts or fewer reducing sugars than cultures grown on SG- or SS-based medium, as determined by pairwise *t*-tests of fitted data from the model The middle black bar at the center of the box indicates the median value, edges of boxes indicate the interquartile range and whiskers indicate minimum and maximum values. Click here for file

Additional file 2 **Supplemental Table 1**. Complete list of isolates tested in this study. All isolates were tested on filter paper, xylan, arabinoxylan, xyloglucan, switchgrass, corn stalk, alfalfa and soybean stem. NYFC, New York Field Crop Pathogen Collection (Gary Bergstrom, Cornell University, Ithaca, NY, USA); CPP, Cornell Department of Plant Pathology and Plant-Microbe Biology Culture Collection (David Kalb, Cornell University, Ithaca, NY, USA); KO, Kerry O'Donnell (USDA-ARS, Peoria, IL, USA); DG, David Geiser (Penn State University, State College, PA, USA); TZ, Tom Zitter (Cornell University, Ithaca, NY, USA); GH, Gary Harman (Cornell University, Geneva, NY, USA). A single asterisk indicates isolates used to determine effect of growth media. Click here for file

Additional file 3 **Supplemental Figure 2**. Ranking of 156 species for hydrolysis of eight polysaccharides and plant cell walls. Response is presented in μM reducing sugar present in hydrolysate. Species are ranked by median values, indicated by center black bars. The edges of each box indicate the interquartile range. Whiskers indicate minimum and maximum values or 1.5 times the interquartile range of the data in the case of outliers which are represented by '*'. Vertical dashed grey lines indicate minimum and maximum species medians for each substrate. Click here for file

Additional file 4 **Supplemental Figure 3**. Hierarchical clustering of complete dataset, excluding *Trichoderma reesei*. Heatmap showing mean activities and clustering of 155 species of plant pathogenic and non-pathogenic fungi when assayed for hydrolysis of eight polysaccharide substrates [XG, xyloglucan (from tamarind); FP, filter paper; AL, alfalfa; SS, soybean stems; SG, switchgrass; CS, corn stalks; AXW, arabinoxylan (from wheat); XY, xylan (from birch)]. *T. reesei *RUT-C30 was excluded from this analysis because of its unusual hydrolytic activities. Negative estimations of reducing sugars were adjusted to zero and data were standardized within substrates by subtracting the substrate mean and dividing by the standard deviation. Dendrogram and ordering was determined using the distance matrix computation (dist) and hierarchical clustering (hclust) functions in R. Red colors indicate values greater than the substrate mean, while blue colors indicate values less than the mean. Column *Z*-score and color intensity indicate how many standard deviations the species mean is from the substrate mean. Click here for file

## References

[B1] Merino S Cherry J Progress and challenges in enzyme development for biomass utilization Biofuels 2007 108 95 120 full_text 10.1007/10_2007_066 17594064

[B2] Committee on a New Biology for the 21st Century, National Research Council A New Biology for the 21st Century 2009 Washington, DC: The National Academies Press

[B3] Sarkar P Bosneaga E Auer M Plant cell walls throughout evolution: towards a molecular understanding of their design principles J Exp Bot 2009 60 3615 3635 10.1093/jxb/erp245 19687127

[B4] Vogel J Unique aspects of the grass cell wall Curr Opin Plant Biol 2008 11 301 307 10.1016/j.pbi.2008.03.002 18434239

[B5] Carpita NC Structure and biogenesis of the cell walls of grasses Annu Rev Plant Physiol Plant Mol Biol 1996 47 445 476 10.1146/annurev.arplant.47.1.445 15012297

[B6] Benoit I Danchin E Bleichrodt R de Vries R Biotechnological applications and potential of fungal feruloyl esterases based on prevalence, classification and biochemical diversity Biotechnol Lett 2008 30 387 396 10.1007/s10529-007-9564-6 17973091

[B7] Henrissat B A classification of glycosyl hydrolases based on amino-acid-sequence similarities Biochem J 1991 280 309 316 1747104 10.1042/bj2800309 PMC1130547

[B8] Henrissat B Teeri T Warren R A scheme for designating enzymes that hydrolyse the polysaccharides in the cell walls of plants FEBS Lett 1998 425 352 354 10.1016/S0014-5793(98)00265-8 9559678

[B9] Henrissat B Bairoch A New families in the classification of glycosyl hydrolases based on amino acid sequence similarities Biochem J 1993 293 Pt 3 781 788 8352747 10.1042/bj2930781 PMC1134435

[B10] Henrissat B Bairoch A Updating the sequence-based classification of glycosyl hydrolases Biochem J 1996 316 695 696 8687420 10.1042/bj3160695 PMC1217404

[B11] Cantarel BL Coutinho PM Rancurel C Bernard T Lombard V Henrissat B The Carbohydrate-Active EnZymes database (CAZy): an expert resource for glycogenomics Nucleic Acids Res 2009 37 D233 D238 10.1093/nar/gkn663 18838391 PMC2686590

[B12] Martinez D Berka RM Henrissat B Saloheimo M Arvas M Baker SE Chapman J Chertkov O Coutinho PM Cullen D Genome sequencing and analysis of the biomass-degrading fungus *Trichoderma reesei *(syn. *Hypocrea jecorina*) Nat Biotech 2008 26 553 560 10.1038/nbt1403 18454138

[B13] Ghosh A Ghosh BK Trimino-Vazquez H Eveleigh DE Montenecourt BS Cellulase secretion from a hyper-cellulolytic mutant of *Trichoderma reesei *Rut-C30 Arch Microbiol 1984 140 126 133 10.1007/BF00454914

[B14] Ghosh A Al-Rabiai S Ghosh B Trimiño-Vazquez H Eveleigh D Montenecourt B Increased endoplasmic reticulum content of a mutant of *Trichoderma reesei *(RUT-C30) in relation to cellulase synthesis Enzyme Microb Technol 1982 4 110 113 10.1016/0141-0229(82)90093-X

[B15] Sheir-Neiss G Montenecourt BS Characterization of the secreted cellulases of *Trichoderma reesei *wild type and mutants during controlled fermentations Appl Microbiol Biotechnol 1984 20 46 53 10.1007/BF00254645

[B16] Gao D Chundawat S Liu T Hermanson S Gowda K Brumm P Dale B Balan V Strategy for identification of novel fungal and bacterial glycosyl hydrolase hybrid mixtures that can efficiently saccharify pretreated lignocellulosic biomass Bioenerg Res 2010 3 67 81 10.1007/s12155-009-9066-6

[B17] Rosgaard L Pedersen S Cherry JR Harris P Meyer AS Efficiency of new fungal cellulase systems in boosting enzymatic degradation of barley straw lignocellulose Biotechnol Prog 2006 22 493 498 10.1021/bp050361o 16599567

[B18] Harris PV Welner D McFarland KC Re E Navarro Poulsen J Brown K Salbo R Ding H Vlasenko E Merino S Stimulation of lignocellulosic biomass hydrolysis by proteins of glycoside hydrolase family 61: Structure and function of a large, enigmatic family Biochemistry 2010 49 3305 3316 10.1021/bi100009p 20230050

[B19] Vaaje-Kolstad G Westereng B Horn SJ Liu Z Zhai H Sørlie M Eijsink VGH An oxidative enzyme boosting the enzymatic conversion of recalcitrant polysaccharides Science 2010 330 219 222 10.1126/science.1192231 20929773

[B20] Spanu PD Abbott JC Amselem J Burgis TA Soanes DM Stüber K Loren van Themaat EV Brown JKM Butcher SA Gurr SJ Genome expansion and gene loss in powdery mildew fungi reveal tradeoffs in extreme parasitism Science 2010 330 1543 1546 10.1126/science.1194573 21148392

[B21] Anderson AJ Extracellular enzymes produced by *Colletotrichum lindemuthianum *and *Helminthosporium maydis *during growth on isolated bean and corn cell walls Phytopathol 1978 68 1585 1589 10.1094/Phyto-68-1585

[B22] Albersheim P Anderson-Prouty AJ Carbohydrates, proteins, cell surfaces, and the biochemistry of pathogenesis Annu Rev Plant Physiol 1975 26 31 52 10.1146/annurev.pp.26.060175.000335

[B23] De Lorenzo G D'Ovidio R Cervone F The role of polygalacturonase-inhibiting proteins (PGIPs) in defense against pathogenic fungi Annu Rev Phytopathol 2001 39 313 335 10.1146/annurev.phyto.39.1.313 11701868

[B24] Kemp G Bergmann CW Clay R Van der Westhuizen AJ Pretorius ZA Isolation of a polygalacturonase-inhibiting protein (PGIP) from wheat Mol Plant Microbe Interac 2003 16 955 961 10.1094/MPMI.2003.16.11.955 14601663

[B25] Jang S Lee B Kim C Kim S Yim J Han J Lee S Kim S An G The OsFOR1 gene encodes a polygalacturonase-inhibiting protein (PGIP) that regulates floral organ number in rice Plant Mol Bio 2003 53 357 372 10.1023/B:PLAN.0000006940.89955.f1 14750524

[B26] Goesaert H Elliott G Kroon PA Gebruers K Courtin CM Robben J Delcour JA Juge N Occurrence of proteinaceous endoxylanase inhibitors in cereals Biochim Biophys Acta, Proteins Proteomics 2004 1696 193 202 10.1016/j.bbapap.2003.08.015 14871660

[B27] Juge N Svensson B Proteinaceous inhibitors of carbohydrate-active enzymes in cereals: implication in agriculture, cereal processing and nutrition J Sci Food Agric 2006 86 1573 1586 10.1002/jsfa.2454

[B28] Croes E Gebruers K Robben J Noben J Samyn B Debyser G Beeumen JV Delcour JA Courtin CM Variability of polymorphic families of three types of xylanase inhibitors in the wheat grain proteome Proteomics 2008 8 1692 1705 10.1002/pmic.200700813 18340629

[B29] Agrios GN Plant Pathology 2005 5 Academic Press

[B30] Cooper R Longman D Campbell A Henry M Lees P Enzymic adaptation of cereal pathogens to the monocotyledonous primary wall Physiol Mol Plant Path 1988 32 33 47 10.1016/S0885-5765(88)80004-3

[B31] Zalewska-Sobczak J Sequential secretion of cell wall degrading enzymes by *Botrytis fabae *and *Fusarium avenaceum *during growth on host and non-host plants [broad bean, rye] Biochem Physiol Pfl 1985 180 169 175

[B32] Paper JM Scott-Craig JS Adhikari ND Cuomo CA Walton JD Comparative proteomics of extracellular proteins *in vitro *and *in planta *from the pathogenic fungus *Fusarium graminearum* Proteomics 2007 7 3171 3183 10.1002/pmic.200700184 17676664

[B33] ten Have A Tenberge KB Benen JAE Tudzynski P Visser J van Kan JAL Kempken F The contribution of cell wall degrading enzymes to pathogenesis of fungal plant pathogens The Mycota XI, Agricultural Applications 2002 Berlin: Springer 341 358

[B34] Douaiher M Nowak E Durand R Halama P Reignault P Correlative analysis of *Mycosphaerella graminicola *pathogenicity and cell wall-degrading enzymes produced *in vitro*: the importance of xylanase and polygalacturonase Plant Pathol 2007 56 79 86 10.1111/j.1365-3059.2006.01460.x

[B35] Siah A Deweer C Duyme F Sanssené J Durand R Halama P Reignault P Correlation of *in planta *endo-beta-1,4-xylanase activity with the necrotrophic phase of the hemibiotrophic fungus *Mycosphaerella graminicola* Plant Pathol 2010 59 661 670 10.1111/j.1365-3059.2010.02303.x

[B36] Selig MJ Knoshaug EP Adney WS Himmel ME Decker SR Synergistic enhancement of cellobiohydrolase performance on pretreated corn stover by addition of xylanase and esterase activities Bioresource Technol 2008 99 4997 5005 10.1016/j.biortech.2007.09.064 18006303

[B37] Tabka M Herpoël-Gimbert I Monod F Asther M Sigoillot J Enzymatic saccharification of wheat straw for bioethanol production by a combined cellulase xylanase and feruloyl esterase treatment Enzyme Microb Tech 2006 39 897 902 10.1016/j.enzmictec.2006.01.021

[B38] Banerjee G Car S Scott-Craig JS Borrusch MS Bongers M Walton JD Synthetic multi-component enzyme mixtures for deconstruction of lignocellulosic biomass Bioresource Technol 2010 101 9097 9105 10.1016/j.biortech.2010.07.028 20678930

[B39] Shaner G Stromberg EL Lacy GH Barker KR Pirone TP Nomenclature and concepts of pathogenicity and virulence Annu Rev Phytopathol 1992 30 47 66 10.1146/annurev.py.30.090192.000403 18643770

[B40] Reignault P Valette-Collet O Boccara M The importance of fungal pectinolytic enzymes in plant invasion, host adaptability and symptom type Eur J Plant Pathol 2008 120 1 11 10.1007/s10658-007-9184-y

[B41] Tonukari NJ Scott-Craig JS Walton JD The *Cochliobolus carbonum *SNF1 gene is required for cell wall-degrading enzyme expression and virulence on maize Plant Cell 2000 12 237 247 10.1105/tpc.12.2.237 10662860 PMC139761

[B42] Scott-Craig JS Panaccione DG Cervone F Walton JD Endopolygalacturonase is not required for pathogenicity of *Cochliobolus carbonum *on maize Plant Cell 1990 2 1191 1200 10.1105/tpc.2.12.1191 2152162 PMC159966

[B43] Banerjee G Scott-Craig J Walton J Improving enzymes for biomass conversion: a basic research perspective Bioenerg Res 2010 3 82 92 10.1007/s12155-009-9067-5

[B44] King B Donnelly M Bergstrom G Walker L Gibson D An optimized microplate assay system for quantitative evaluation of plant cell wall-degrading enzyme activity of fungal culture extracts Biotechnol Bioeng 2009 102 1033 1044 10.1002/bit.22151 18973283

[B45] Decker S Adney W Jennings E Vinzant T Himmel M Automated filter paper assay for determination of cellulase activity Appl Biochem Biotechnol 2003 105 689 703 10.1385/ABAB:107:1-3:689 12721448

[B46] Xiao Z Storms R Tsang A Microplate-based filter paper assay to measure total cellulase activity Biotechnol Bioeng 2004 88 832 837 10.1002/bit.20286 15459905

[B47] Xiao Z Storms R Tsang A Microplate-based carboxymethylcellulose assay for endoglucanase activity Anal Biochem 2005 342 176 178 10.1016/j.ab.2005.01.052 15958198

[B48] Ghose T Measurement of cellulase activities Pure Appl Chem 1987 59 257 268 10.1351/pac198759020257

