# Supplementary material for: Arsenal of plant cell wall degrading enzymes reflects host preference among plant pathogenic fungi
Source: Biotechnol Biofuels. 2011 Feb 16;4:4. doi: 10.1186/1754-6834-4-4 (PMC3051899; doi:10.1186/1754-6834-4-4)
Supplement: Additional file 1 — Supplemental Figure 1. Interactions among hosts, growth media, and substrates. Fitted values from mixed-effect model on activity standardized within substrates. Data are from six dicot (d) pathogens and six monocot (m) pathogens grown on Avicel (A), switchgrass (SG) and soybean-stem (SS) supplemented minimal media. The lower case letter on the x-axis label indicates pathogen host (d, m) and the upper case letters indicate growth media (A, SG, SS). Each set of plots is for nine different substrates (FP, CMC, XY, AXW, XG, AL, SS, CS, SG). The effects of host, media and substrate, as well as their interactions, were treated as fixed effects and isolate was treated as a random effect. The third order interaction of host*substrate*medium was significant (P = 0.0135), as was the second order interaction of medium*substrate (P = 0.0184) and the primary effect of substrate (P = 0.0009). For all assay substrates, extracts from fungi grown on the Avicel-based medium released either comparable amounts or fewer reducing sugars than cultures grown on SG- or SS-based medium, as determined by pairwise t-tests of fitted data from the model The middle black bar at the center of the box indicates the median value, edges of boxes indicate the interquartile range and whiskers indicate minimum and maximum values. [file 1754-6834-4-4-S1.PDF]

Figure S1 – Interactions among hosts, growth media, and substrates

Fitted values from mixed-effect model on activity standardized within substrates. Data are from six dicot (d) pathogens and six monocot (m) pathogens grown on Avicel (A), switchgrass (SG), and soybean-stem (SS) supplemented minimal media. The lower case letter on the x-axis label indicates pathogen host (d, m) and the upper case letters indicate growth media (A, SG, SS). Each set of plots is for nine different substrates (FP, CMC, XY, AXW, XG, AL, SS, CS, SG). The effects of host, media, and substrate as well as their interactions were treated as fixed effects, and isolate was treated as a random effect. The third order interaction of host*substrate*medium was significant ($p = 0.0135$), as was the second order interaction of medium*substrate ($p = 0.0184$) and the primary effect of substrate ($p = 0.0009$). For all assay substrates, extracts from fungi grown on the Avicel-based medium released either comparable amounts or fewer reducing sugars than cultures grown on SG- or SS-based medium, as determined by pairwise t-tests of fitted data from the model The middle black bar at the center of the box indicates the median value, edges of boxes indicate the interquartile range, and whiskers indicate minimum and maximum values.

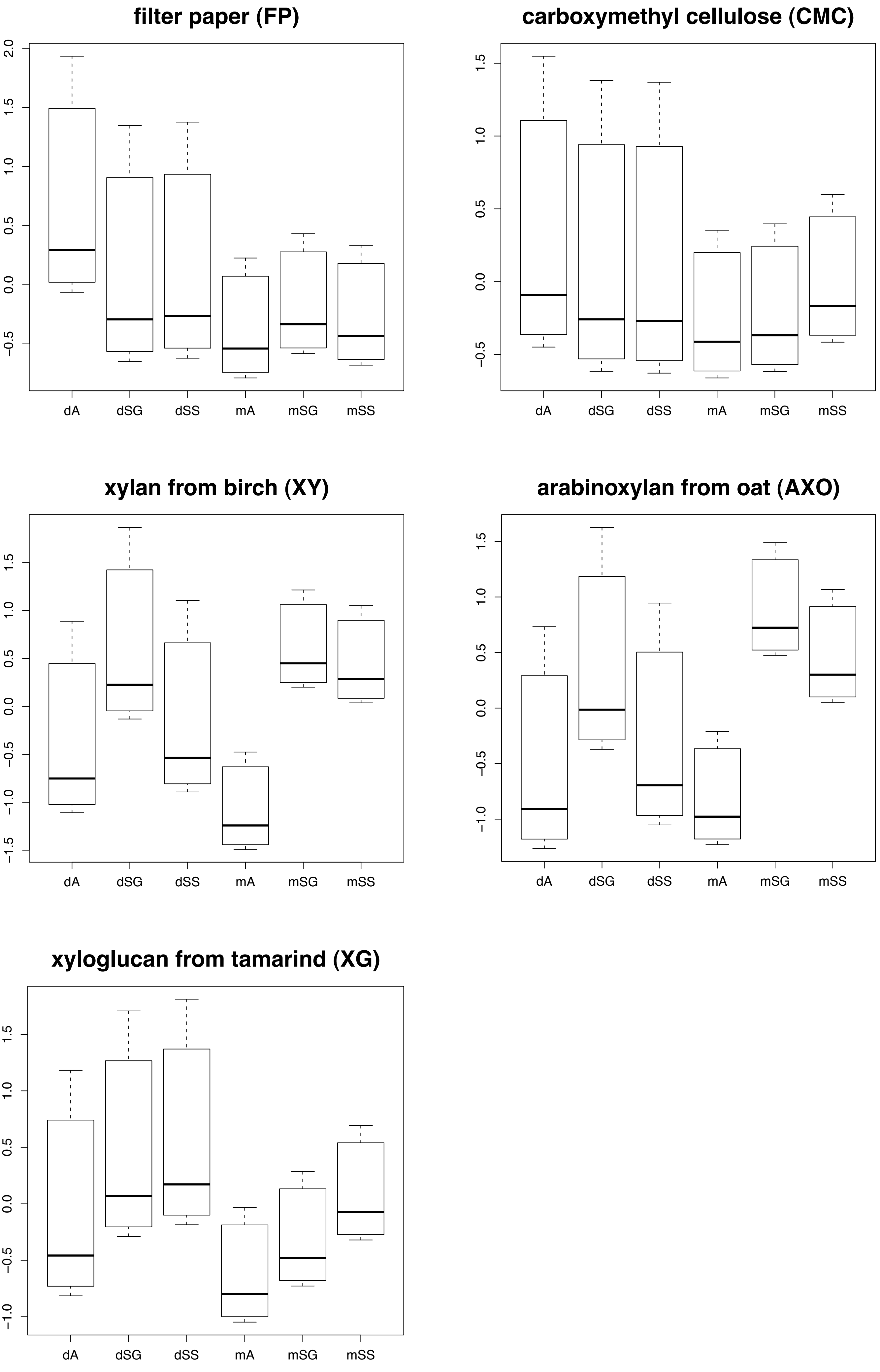
filter paper (FP)
-0.5 0.0 0.5 1.0 1.5 2.0
dA dSG dSS mA mSG mSS
carboxymethyl cellulose (CMC)
-0.5 0.0 0.5 1.0 1.5
dA dSG dSS mA mSG mSS
xylan from birch (XY)
-1.5 -1.0 -0.5 0.0 0.5 1.0 1.5
dA dSG dSS mA mSG mSS
arabinoxylan from oat (AXO)
-1.0 -0.5 0.0 0.5 1.0 1.5
dA dSG dSS mA mSG mSS
xyloglucan from tamarind (XG)
-1.0 -0.5 0.0 0.5 1.0 1.5
dA dSG dSS mA mSG mSS

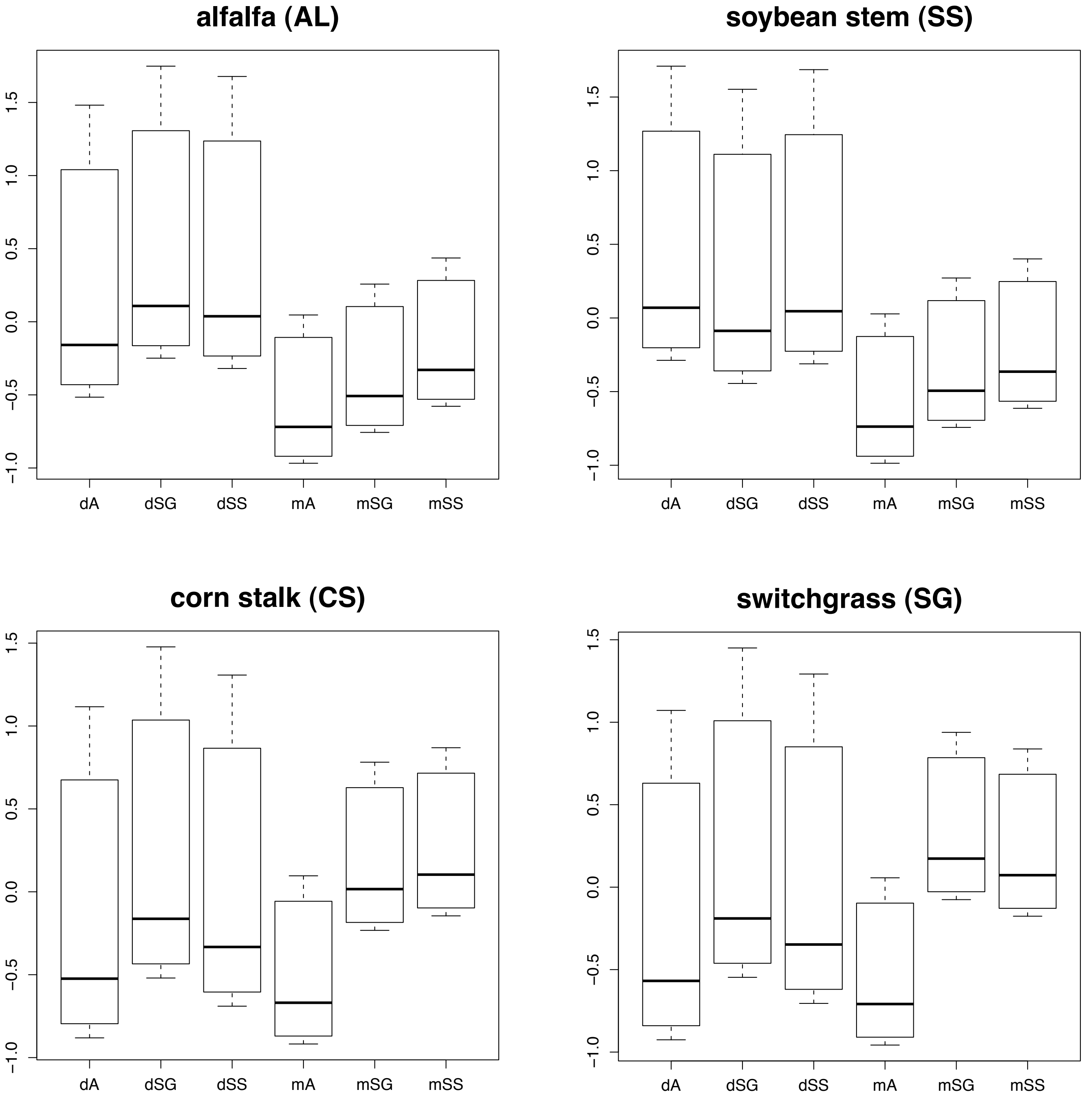

alfalfa (AL)
soybean stem (SS)
corn stalk (CS)
switchgrass (SG)
dA
dSG
dSS
mA
mSG
mSS
−1.0
−0.5
0.0
0.5
1.0
1.5
