# Supplementary material for: Arsenal of plant cell wall degrading enzymes reflects host preference among plant pathogenic fungi
Source: Biotechnol Biofuels. 2011 Feb 16;4:4. doi: 10.1186/1754-6834-4-4 (PMC3051899; doi:10.1186/1754-6834-4-4)
Supplement: Additional file 3 — Supplemental Figure 2. Ranking of 156 species for hydrolysis of eight polysaccharides and plant cell walls. Response is presented in μM reducing sugar present in hydrolysate. Species are ranked by median values, indicated by center black bars. The edges of each box indicate the interquartile range. Whiskers indicate minimum and maximum values or 1.5 times the interquartile range of the data in the case of outliers which are represented by '*'. Vertical dashed grey lines indicate minimum and maximum species medians for each substrate. [file 1754-6834-4-4-S3.PDF]

Figure S2 - Ranking of 156 species for hydrolysis of eight polysaccharides and plant cell walls.

Response is presented in μM reducing sugar present in hydrolysate. Species are ranked by median values, indicated by center black bars. The edges of each box indicate the interquartile range. Whiskers indicate minimum and maximum values or 1.5 times the interquartile range of the data in the case of outliers which are represented by "*". Vertical dashed grey lines indicate minimum and maximum species medians for each substrate.

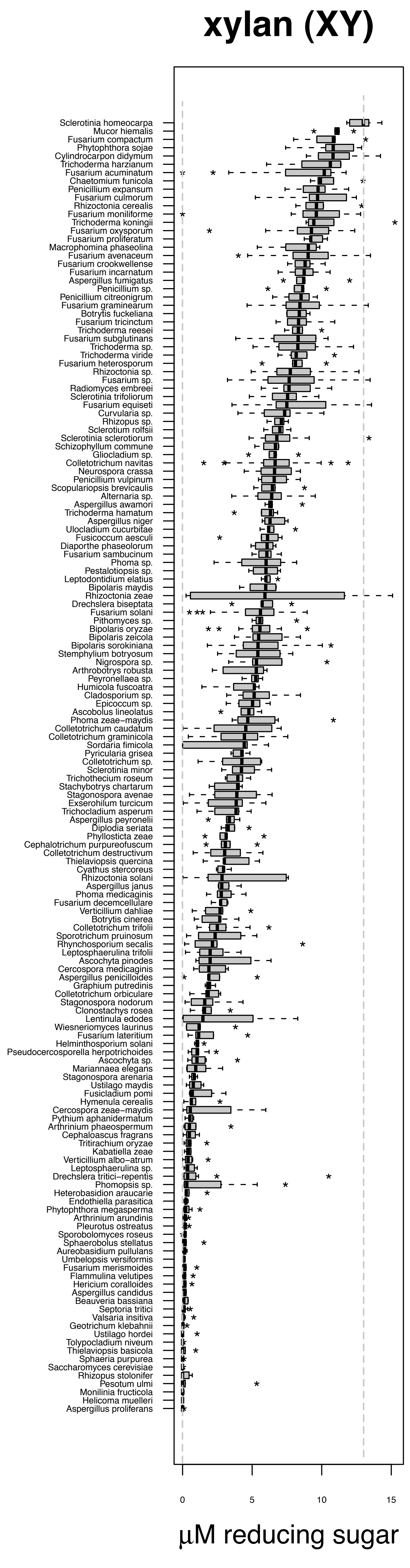
xylan (XY)
Sclerotinia homeocarpa
Mucor hiemalis
Fusarium compactum
Phytophthora sojae
Cylindrocarpon didymum
Trichoderma harzianum
Fusarium acuminatum
Chaetomium funicola
Penicillium expansum
Fusarium culmorum
Rhizoctonia cerealis
Fusarium moniliforme
Trichoderma koningii
Fusarium oxysporum
Fusarium proliferatum
Macrophomina phaseolina
Fusarium avenaceum
Fusarium crookwellense
Fusarium incarnatum
Aspergillus fumigatus
Penicillium sp.
Penicillium citreonigrum
Fusarium graminearum
Botrytis fuckeliana
Fusarium tricinctum
Trichoderma reesei
Fusarium subglutinans
Trichoderma sp.
Trichoderma viride
Fusarium heterosporum
Rhizoctonia sp.
Fusarium sp.
Radiomyces embreei
Sclerotinia trifoliorum
Fusarium equiseti
Curvularia sp.
Rhizopus sp.
Sclerotium rolfsii
Sclerotinia sclerotiorum
Schizophyllum commune
Gliocladium sp.
Colletotrichum navitas
Neurospora crassa
Penicillium vulpinum
Scopulariopsis brevicaulis
Alternaria sp.
Aspergillus awamori
Trichoderma hamatum
Aspergillus niger
Ulocladium cucurbitae
Fusicoccum aesculi
Diaporthe phaseolorum
Fusarium sambucinum
Phoma sp.
Pestalotiopsis sp.
Leptodontidium elatius
Bipolaris maydis
Rhizoctonia zeae
Drechslera biseptata
Fusarium solani
Pithomyces sp.
Bipolaris oryzae
Bipolaris zeicola
Bipolaris sorokiniana
Stemphylium botryosum
Nigrospora sp.
Arthrobotrys robusta
Peyronellaea sp.
Humicola fuscoatra
Cladosporium sp.
Epicoccum sp.
Ascobolus lineolatus
Phoma zeae−maydis
Colletotrichum caudatum
Colletotrichum graminicola
Sordaria fimicola
Pyricularia grisea
Colletotrichum sp.
Sclerotinia minor
Trichothecium roseum
Stachybotrys chartarum
Stagonospora avenae
Exserohilum turcicum
Trichocladium asperum
Aspergillus peyronelii
Diplodia seriata
Phyllosticta zeae
Cephalotrichum purpureofuscum
Colletotrichum destructivum
Thielaviopsis quercina
Cyathus stercoreus
Rhizoctonia solani
Aspergillus janus
Phoma medicaginis
Fusarium decemcellulare
Verticillium dahliae
Botrytis cinerea
Colletotrichum trifolii
Sporotrichum pruinosum
Rhynchosporium secalis
Leptosphaerulina trifolii
Ascochyta pinodes
Cercospora medicaginis
Aspergillus penicilloides
Graphium putredinis
Colletotrichum orbiculare
Stagonospora nodorum
Clonostachys rosea
Lentinula edodes
Wiesneriomyces laurinus
Fusarium lateritium
Helminthosporium solani
Pseudocercosporella herpotrichoides
Ascochyta sp.
Mariannaea elegans
Stagonospora arenaria
Ustilago maydis
Fusicladium pomi
Hymenula cerealis
Cercospora zeae−maydis
Pythium aphanidermatum
Arthrinium phaeospermum
Cephaloascus fragrans
Tritirachium oryzae
Kabatiella zeae
Verticillium albo−atrum
Leptosphaerulina sp.
Drechslera tritici−repentis
Phomopsis sp.
Heterobasidion araucarie
Endothiella parasitica
Phytophthora megasperma
Arthrinium arundinis
Pleurotus ostreatus
Sporobolomyces roseus
Sphaerobolus stellatus
Aureobasidium pullulans
Umbelopsis versiformis
Fusarium merismoides
Flammulina velutipes
Hericium coralloides
Aspergillus candidus
Beauveria bassiana
Septoria tritici
Valsaria insitiva
Geotrichum klebahnii
Ustilago hordei
Tolypocladium niveum
Thielaviopsis basicola
Sphaeria purpurea
Saccharomyces cerevisiae
Rhizopus stolonifer
Pesotum ulmi
Monilinia fructicola
Helicoma muelleri
Aspergillus proliferans
0
5
10
15
μM reducing sugar

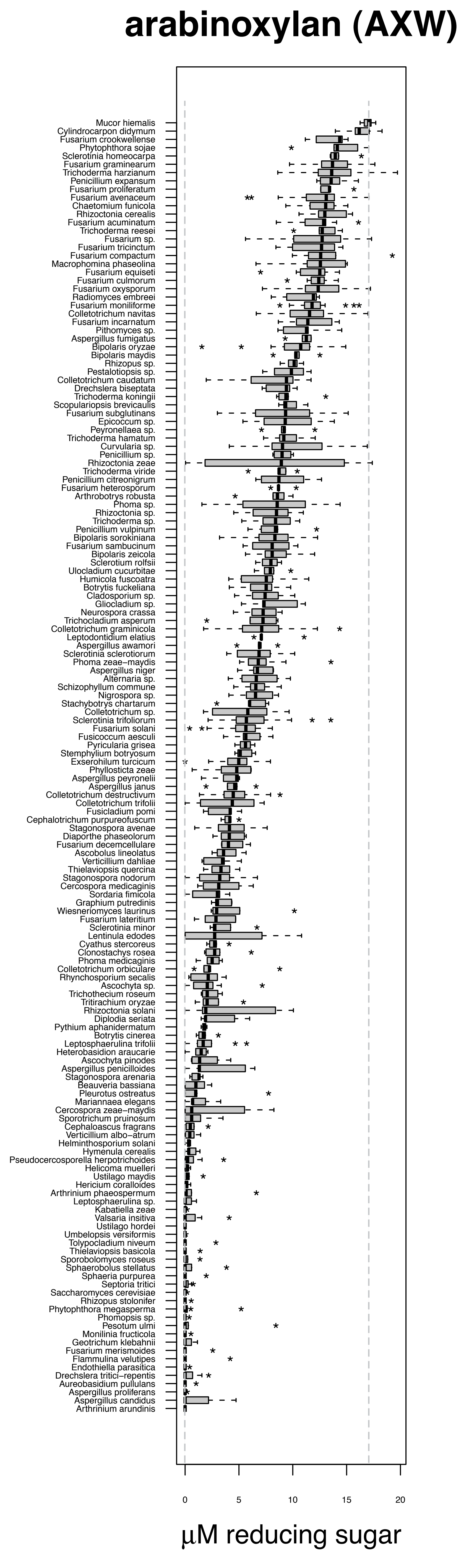
arabinoxylan (AXW)
Mucor hiemalis
Cylindrocarpon didymum
Fusarium crookwellense
Phytophthora sojae
Sclerotinia homeocarpa
Fusarium graminearum
Trichoderma harzianum
Penicillium expansum
Fusarium proliferatum
Fusarium avenaceum
Chaetomium funicola
Rhizoctonia cerealis
Fusarium acuminatum
Trichoderma reesei
Fusarium sp.
Fusarium tricinctum
Fusarium compactum
Macrophomina phaseolina
Fusarium equiseti
Fusarium culmorum
Fusarium oxysporum
Radiomyces embreei
Fusarium moniliforme
Colletotrichum navitas
Fusarium incarnatum
Pithomyces sp.
Aspergillus fumigatus
Bipolaris oryzae
Bipolaris maydis
Rhizopus sp.
Pestalotiopsis sp.
Colletotrichum caudatum
Drechslera biseptata
Trichoderma koningii
Scopulariopsis brevicaulis
Fusarium subglutinans
Epicoccum sp.
Peyronellaea sp.
Trichoderma hamatum
Curvularia sp.
Penicillium sp.
Rhizoctonia zeae
Trichoderma viride
Penicillium citreonigrum
Fusarium heterosporum
Arthrobotrys robusta
Phoma sp.
Rhizoctonia sp.
Trichoderma sp.
Penicillium vulpinum
Bipolaris sorokiniana
Fusarium sambucinum
Bipolaris zeicola
Sclerotium rolfsii
Ulocladium cucurbitae
Humicola fuscoatra
Botrytis fuckeliana
Cladosporium sp.
Gliocladium sp.
Neurospora crassa
Trichocladium asperum
Colletotrichum graminicola
Leptodontidium elatius
Aspergillus awamori
Sclerotinia sclerotiorum
Phoma zeae−maydis
Aspergillus niger
Alternaria sp.
Schizophyllum commune
Nigrospora sp.
Stachybotrys chartarum
Colletotrichum sp.
Sclerotinia trifoliorum
Fusarium solani
Fusicoccum aesculi
Pyricularia grisea
Stemphylium botryosum
Exserohilum turcicum
Phyllosticta zeae
Aspergillus peyronelii
Aspergillus janus
Colletotrichum destructivum
Colletotrichum trifolii
Fusicladium pomi
Cephalotrichum purpureofuscum
Stagonospora avenae
Diaporthe phaseolorum
Fusarium decemcellulare
Ascobolus lineolatus
Verticillium dahliae
Thielaviopsis quercina
Stagonospora nodorum
Cercospora medicaginis
Sordaria fimicola
Graphium putredinis
Wiesneriomyces laurinus
Fusarium lateritium
Sclerotinia minor
Lentinula edodes
Cyathus stercoreus
Clonostachys rosea
Phoma medicaginis
Colletotrichum orbiculare
Rhynchosporium secalis
Ascochyta sp.
Trichothecium roseum
Tritirachium oryzae
Rhizoctonia solani
Diplodia seriata
Pythium aphanidermatum
Botrytis cinerea
Leptosphaerulina trifolii
Heterobasidion araucarie
Ascochyta pinodes
Aspergillus penicilloides
Stagonospora arenaria
Beauveria bassiana
Pleurotus ostreatus
Mariannaea elegans
Cercospora zeae−maydis
Sporotrichum pruinosum
Cephaloascus fragrans
Verticillium albo−atrum
Helminthosporium solani
Hymenula cerealis
Pseudocercosporella herpotrichoides
Helicoma muelleri
Ustilago maydis
Hericium coralloides
Arthrinium phaeospermum
Leptosphaerulina sp.
Kabatiella zeae
Valsaria insitiva
Ustilago hordei
Umbelopsis versiformis
Tolypocladium niveum
Thielaviopsis basicola
Sporobolomyces roseus
Sphaerobolus stellatus
Sphaeria purpurea
Septoria tritici
Saccharomyces cerevisiae
Rhizopus stolonifer
Phytophthora megasperma
Phomopsis sp.
Pesotum ulmi
Monilinia fructicola
Geotrichum klebahnii
Fusarium merismoides
Flammulina velutipes
Endothiella parasitica
Drechslera tritici−repentis
Aureobasidium pullulans
Aspergillus proliferans
Aspergillus candidus
Arthrinium arundinis
0
5
10
15
20
μM reducing sugar

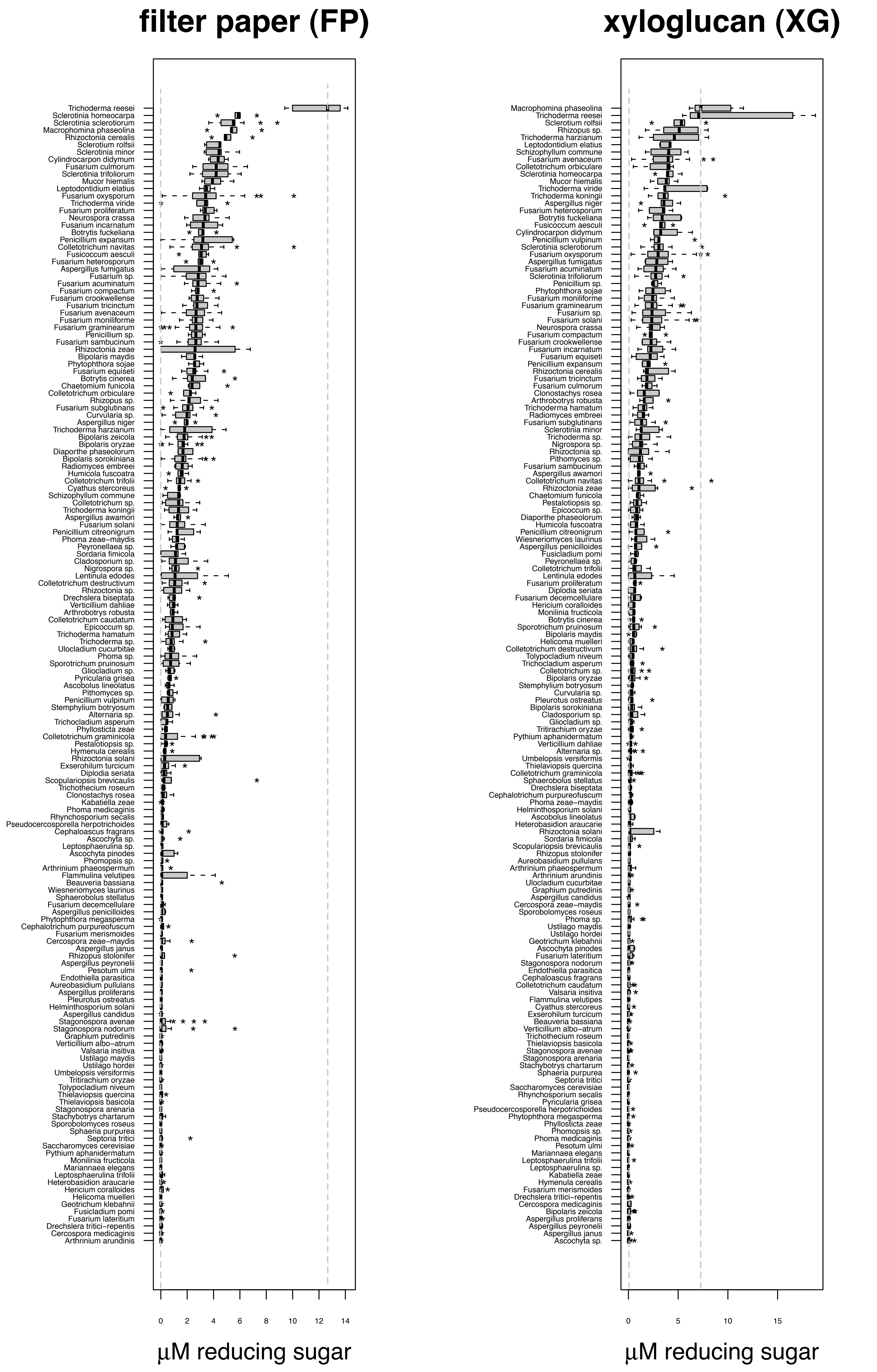
filter paper (FP)
Trichoderma reesei
Sclerotinia homeocarpa
Sclerotinia sclerotiorum
Macrophomina phaseolina
Rhizoctonia cerealis
Sclerotium rolfsii
Sclerotinia minor
Cylindrocarpon didymum
Fusarium culmorum
Sclerotinia trifoliorum
Mucor hiemalis
Leptodontidium elatius
Fusarium oxysporum
Trichoderma viride
Fusarium proliferatum
Neurospora crassa
Fusarium incarnatum
Botrytis fuckeliana
Penicillium expansum
Colletotrichum navitas
Fusicoccum aesculi
Fusarium heterosporum
Aspergillus fumigatus
Fusarium sp.
Fusarium acuminatum
Fusarium compactum
Fusarium crookwellense
Fusarium tricinctum
Fusarium avenaceum
Fusarium moniliforme
Fusarium graminearum
Penicillium sp.
Fusarium sambucinum
Rhizoctonia zeae
Bipolaris maydis
Phytophthora sojae
Fusarium equiseti
Botrytis cinerea
Chaetomium funicola
Colletotrichum orbiculare
Rhizopus sp.
Fusarium subglutinans
Curvularia sp.
Aspergillus niger
Trichoderma harzianum
Bipolaris zeicola
Bipolaris oryzae
Diaporthe phaseolorum
Bipolaris sorokiniana
Radiomyces embreei
Humicola fuscoatra
Colletotrichum trifolii
Cyathus stercoreus
Schizophyllum commune
Colletotrichum sp.
Trichoderma koningii
Aspergillus awamori
Fusarium solani
Penicillium citreonigrum
Phoma zeae−maydis
Peyronellaea sp.
Sordaria fimicola
Cladosporium sp.
Nigrospora sp.
Lentinula edodes
Colletotrichum destructivum
Rhizoctonia sp.
Drechslera biseptata
Verticillium dahliae
Arthrobotrys robusta
Colletotrichum caudatum
Epicoccum sp.
Trichoderma hamatum
Trichoderma sp.
Ulocladium cucurbitae
Phoma sp.
Sporotrichum pruinosum
Gliocladium sp.
Pyricularia grisea
Ascobolus lineolatus
Pithomyces sp.
Penicillium vulpinum
Stemphylium botryosum
Alternaria sp.
Trichocladium asperum
Phyllosticta zeae
Colletotrichum graminicola
Pestalotiopsis sp.
Hymenula cerealis
Rhizoctonia solani
Exserohilum turcicum
Diplodia seriata
Scopulariopsis brevicaulis
Trichothecium roseum
Clonostachys rosea
Kabatiella zeae
Phoma medicaginis
Rhynchosporium secalis
Pseudocercosporella herpotrichoides
Cephaloascus fragrans
Ascochyta sp.
Leptosphaerulina sp.
Ascochyta pinodes
Phomopsis sp.
Arthrinium phaeospermum
Flammulina velutipes
Beauveria bassiana
Wiesneriomyces laurinus
Sphaerobolus stellatus
Fusarium decemcellulare
Aspergillus penicilloides
Phytophthora megasperma
Cephalotrichum purpureofuscum
Fusarium merismoides
Cercospora zeae−maydis
Aspergillus janus
Rhizopus stolonifer
Aspergillus peyronelii
Pesotum ulmi
Endothiella parasitica
Aureobasidium pullulans
Aspergillus proliferans
Pleurotus ostreatus
Helminthosporium solani
Aspergillus candidus
Stagonospora avenae
Stagonospora nodorum
Graphium putredinis
Verticillium albo−atrum
Valsaria insitiva
Ustilago maydis
Ustilago hordei
Umbelopsis versiformis
Tritirachium oryzae
Tolypocladium niveum
Thielaviopsis quercina
Thielaviopsis basicola
Stagonospora arenaria
Stachybotrys chartarum
Sporobolomyces roseus
Sphaeria purpurea
Septoria tritici
Saccharomyces cerevisiae
Pythium aphanidermatum
Monilinia fructicola
Mariannaea elegans
Leptosphaerulina trifolii
Heterobasidion araucarie
Hericium coralloides
Helicoma muelleri
Geotrichum klebahnii
Fusicladium pomi
Fusarium lateritium
Drechslera tritici−repentis
Cercospora medicaginis
Arthrinium arundinis
0
2
4
6
8
10
12
14
µM reducing sugar
xyloglucan (XG)
Macrophomina phaseolina
Trichoderma reesei
Sclerotium rolfsii
Rhizopus sp.
Trichoderma harzianum
Leptodontidium elatius
Schizophyllum commune
Fusarium avenaceum
Colletotrichum orbiculare
Sclerotinia homeocarpa
Mucor hiemalis
Trichoderma viride
Trichoderma koningii
Aspergillus niger
Fusarium heterosporum
Botrytis fuckeliana
Fusicoccum aesculi
Cylindrocarpon didymum
Penicillium vulpinum
Sclerotinia sclerotiorum
Fusarium oxysporum
Aspergillus fumigatus
Fusarium acuminatum
Sclerotinia trifoliorum
Penicillium sp.
Phytophthora sojae
Fusarium moniliforme
Fusarium graminearum
Fusarium sp.
Fusarium solani
Neurospora crassa
Fusarium compactum
Fusarium crookwellense
Fusarium incarnatum
Fusarium equiseti
Penicillium expansum
Rhizoctonia cerealis
Fusarium tricinctum
Fusarium culmorum
Clonostachys rosea
Arthrobotrys robusta
Trichoderma hamatum
Radiomyces embreei
Fusarium subglutinans
Sclerotinia minor
Trichoderma sp.
Nigrospora sp.
Rhizoctonia sp.
Pithomyces sp.
Fusarium sambucinum
Aspergillus awamori
Colletotrichum navitas
Rhizoctonia zeae
Chaetomium funicola
Pestalotiopsis sp.
Epicoccum sp.
Diaporthe phaseolorum
Humicola fuscoatra
Penicillium citreonigrum
Wiesneriomyces laurinus
Aspergillus penicilloides
Fusicladium pomi
Peyronellaea sp.
Colletotrichum trifolii
Lentinula edodes
Fusarium proliferatum
Diplodia seriata
Fusarium decemcellulare
Hericium coralloides
Monilinia fructicola
Botrytis cinerea
Sporotrichum pruinosum
Bipolaris maydis
Helicoma muelleri
Colletotrichum destructivum
Tolypocladium niveum
Trichocladium asperum
Colletotrichum sp.
Bipolaris oryzae
Stemphylium botryosum
Curvularia sp.
Pleurotus ostreatus
Bipolaris sorokiniana
Cladosporium sp.
Gliocladium sp.
Tritirachium oryzae
Pythium aphanidermatum
Verticillium dahliae
Alternaria sp.
Umbelopsis versiformis
Thielaviopsis quercina
Colletotrichum graminicola
Sphaerobolus stellatus
Drechslera biseptata
Cephalotrichum purpureofuscum
Phoma zeae−maydis
Helminthosporium solani
Ascobolus lineolatus
Heterobasidion araucarie
Rhizoctonia solani
Sordaria fimicola
Scopulariopsis brevicaulis
Rhizopus stolonifer
Aureobasidium pullulans
Arthrinium phaeospermum
Arthrinium arundinis
Ulocladium cucurbitae
Graphium putredinis
Aspergillus candidus
Cercospora zeae−maydis
Sporobolomyces roseus
Phoma sp.
Ustilago maydis
Ustilago hordei
Geotrichum klebahnii
Ascochyta pinodes
Fusarium lateritium
Stagonospora nodorum
Endothiella parasitica
Cephaloascus fragrans
Colletotrichum caudatum
Valsaria insitiva
Flammulina velutipes
Cyathus stercoreus
Exserohilum turcicum
Beauveria bassiana
Verticillium albo−atrum
Trichothecium roseum
Thielaviopsis basicola
Stagonospora avenae
Stagonospora arenaria
Stachybotrys chartarum
Sphaeria purpurea
Septoria tritici
Saccharomyces cerevisiae
Rhynchosporium secalis
Pyricularia grisea
Pseudocercosporella herpotrichoides
Phytophthora megasperma
Phyllosticta zeae
Phomopsis sp.
Phoma medicaginis
Pesotum ulmi
Mariannaea elegans
Leptosphaerulina trifolii
Leptosphaerulina sp.
Kabatiella zeae
Hymenula cerealis
Fusarium merismoides
Drechslera tritici−repentis
Cercospora medicaginis
Bipolaris zeicola
Aspergillus proliferans
Aspergillus peyronelii
Aspergillus janus
Ascochyta sp.
0
5
10
15
µM reducing sugar

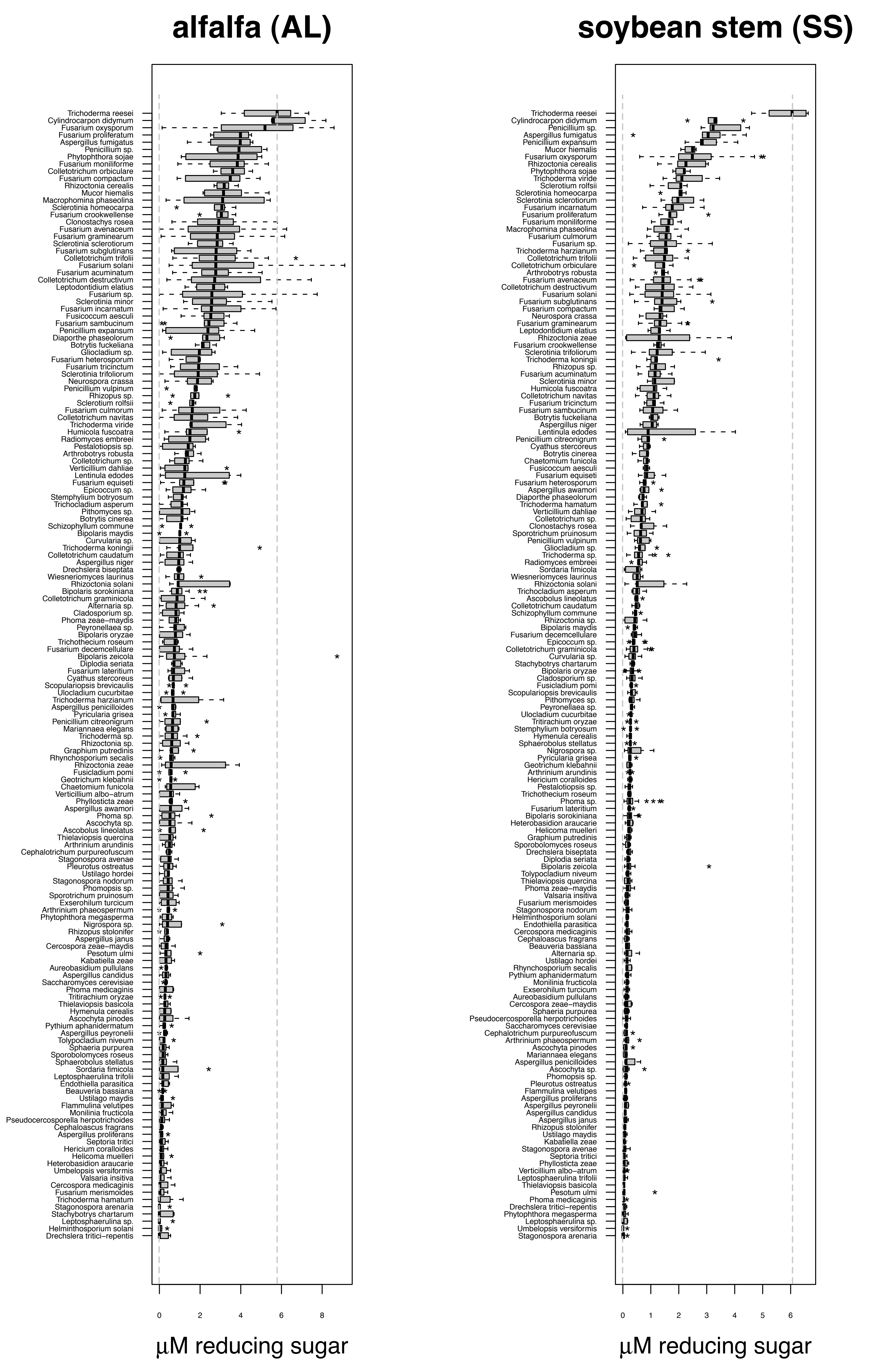

alfalfa (AL)
Trichoderma reesei
Cylindrocarpon didymum
Fusarium oxysporum
Fusarium proliferatum
Aspergillus fumigatus
Penicillium sp.
Phytophthora sojae
Fusarium moniliforme
Colletotrichum orbiculare
Fusarium compactum
Rhizoctonia cerealis
Mucor hiemalis
Macrophomina phaseolina
Sclerotinia homeocarpa
Fusarium crookwellense
Clonostachys rosea
Fusarium avenaceum
Fusarium graminearum
Sclerotinia sclerotiorum
Fusarium subglutinans
Colletotrichum trifolii
Fusarium solani
Fusarium acuminatum
Colletotrichum destructivum
Leptodontidium elatius
Fusarium sp.
Sclerotinia minor
Fusarium incarnatum
Fusicoccum aesculi
Fusarium sambucinum
Penicillium expansum
Diaporthe phaseolorum
Botrytis fuckeliana
Gliocladium sp.
Fusarium heterosporum
Fusarium tricinctum
Sclerotinia trifoliorum
Neurospora crassa
Penicillium vulpinum
Rhizopus sp.
Sclerotium rolfsii
Fusarium culmorum
Colletotrichum navitas
Trichoderma viride
Humicola fuscoatra
Radiomyces embreei
Pestalotiopsis sp.
Arthrobotrys robusta
Colletotrichum sp.
Verticillium dahliae
Lentinula edodes
Fusarium equiseti
Epicoccum sp.
Stemphylium botryosum
Trichocladium asperum
Pithomyces sp.
Botrytis cinerea
Schizophyllum commune
Bipolaris maydis
Curvularia sp.
Trichoderma koningii
Colletotrichum caudatum
Aspergillus niger
Drechslera biseptata
Wiesneriomyces laurinus
Rhizoctonia solani
Bipolaris sorokiniana
Colletotrichum graminicola
Alternaria sp.
Cladosporium sp.
Phoma zeae−maydis
Peyronellaea sp.
Bipolaris oryzae
Trichothecium roseum
Fusarium decemcellulare
Bipolaris zeicola
Diplodia seriata
Fusarium lateritium
Cyathus stercoreus
Scopulariopsis brevicaulis
Ulocladium cucurbitae
Trichoderma harzianum
Aspergillus penicilloides
Pyricularia grisea
Penicillium citreonigrum
Mariannaea elegans
Trichoderma sp.
Rhizoctonia sp.
Graphium putredinis
Rhynchosporium secalis
Rhizoctonia zeae
Fusicladium pomi
Geotrichum klebahnii
Chaetomium funicola
Verticillium albo−atrum
Phyllosticta zeae
Aspergillus awamori
Phoma sp.
Ascochyta sp.
Ascobolus lineolatus
Thielaviopsis quercina
Arthrinium arundinis
Cephalotrichum purpureofuscum
Stagonospora avenae
Pleurotus ostreatus
Ustilago hordei
Stagonospora nodorum
Phomopsis sp.
Sporotrichum pruinosum
Exserohilum turcicum
Arthrinium phaeospermum
Phytophthora megasperma
Nigrospora sp.
Rhizopus stolonifer
Aspergillus janus
Cercospora zeae−maydis
Pesotum ulmi
Kabatiella zeae
Aureobasidium pullulans
Aspergillus candidus
Saccharomyces cerevisiae
Phoma medicaginis
Tritirachium oryzae
Thielaviopsis basicola
Hymenula cerealis
Ascochyta pinodes
Pythium aphanidermatum
Aspergillus peyronelii
Tolypocladium niveum
Sphaeria purpurea
Sporobolomyces roseus
Sphaerobolus stellatus
Sordaria fimicola
Leptosphaerulina trifolii
Endothiella parasitica
Beauveria bassiana
Ustilago maydis
Flammulina velutipes
Monilinia fructicola
Pseudocercosporella herpotrichoides
Cephaloascus fragrans
Aspergillus proliferans
Septoria tritici
Hericium coralloides
Helicoma muelleri
Heterobasidion araucarie
Umbelopsis versiformis
Valsaria insitiva
Cercospora medicaginis
Fusarium merismoides
Trichoderma hamatum
Stagonospora arenaria
Stachybotrys chartarum
Leptosphaerulina sp.
Helminthosporium solani
Drechslera tritici−repentis
0
2
4
6
8
µM reducing sugar
soybean stem (SS)
Trichoderma reesei
Cylindrocarpon didymum
Penicillium sp.
Aspergillus fumigatus
Penicillium expansum
Mucor hiemalis
Fusarium oxysporum
Rhizoctonia cerealis
Phytophthora sojae
Trichoderma viride
Sclerotium rolfsii
Sclerotinia homeocarpa
Sclerotinia sclerotiorum
Fusarium incarnatum
Fusarium proliferatum
Fusarium moniliforme
Macrophomina phaseolina
Fusarium culmorum
Fusarium sp.
Trichoderma harzianum
Colletotrichum trifolii
Colletotrichum orbiculare
Arthrobotrys robusta
Fusarium avenaceum
Colletotrichum destructivum
Fusarium solani
Fusarium subglutinans
Fusarium compactum
Neurospora crassa
Fusarium graminearum
Leptodontidium elatius
Rhizoctonia zeae
Fusarium crookwellense
Sclerotinia trifoliorum
Trichoderma koningii
Rhizopus sp.
Fusarium acuminatum
Sclerotinia minor
Humicola fuscoatra
Colletotrichum navitas
Fusarium tricinctum
Fusarium sambucinum
Botrytis fuckeliana
Aspergillus niger
Lentinula edodes
Penicillium citreonigrum
Cyathus stercoreus
Botrytis cinerea
Chaetomium funicola
Fusicoccum aesculi
Fusarium equiseti
Fusarium heterosporum
Aspergillus awamori
Diaporthe phaseolorum
Trichoderma hamatum
Verticillium dahliae
Colletotrichum sp.
Clonostachys rosea
Sporotrichum pruinosum
Penicillium vulpinum
Gliocladium sp.
Trichoderma sp.
Radiomyces embreei
Sordaria fimicola
Wiesneriomyces laurinus
Rhizoctonia solani
Trichocladium asperum
Ascobolus lineolatus
Colletotrichum caudatum
Schizophyllum commune
Rhizoctonia sp.
Bipolaris maydis
Fusarium decemcellulare
Epicoccum sp.
Colletotrichum graminicola
Curvularia sp.
Stachybotrys chartarum
Bipolaris oryzae
Cladosporium sp.
Fusicladium pomi
Scopulariopsis brevicaulis
Pithomyces sp.
Peyronellaea sp.
Ulocladium cucurbitae
Tritirachium oryzae
Stemphylium botryosum
Hymenula cerealis
Sphaerobolus stellatus
Nigrospora sp.
Pyricularia grisea
Geotrichum klebahnii
Arthrinium arundinis
Hericium coralloides
Pestalotiopsis sp.
Trichothecium roseum
Phoma sp.
Fusarium lateritium
Bipolaris sorokiniana
Heterobasidion araucarie
Helicoma muelleri
Graphium putredinis
Sporobolomyces roseus
Drechslera biseptata
Diplodia seriata
Bipolaris zeicola
Tolypocladium niveum
Thielaviopsis quercina
Phoma zeae−maydis
Valsaria insitiva
Fusarium merismoides
Stagonospora nodorum
Helminthosporium solani
Endothiella parasitica
Cercospora medicaginis
Cephaloascus fragrans
Beauveria bassiana
Alternaria sp.
Ustilago hordei
Rhynchosporium secalis
Pythium aphanidermatum
Monilinia fructicola
Exserohilum turcicum
Aureobasidium pullulans
Cercospora zeae−maydis
Sphaeria purpurea
Pseudocercosporella herpotrichoides
Saccharomyces cerevisiae
Cephalotrichum purpureofuscum
Arthrinium phaeospermum
Ascochyta pinodes
Mariannaea elegans
Aspergillus penicilloides
Ascochyta sp.
Phomopsis sp.
Pleurotus ostreatus
Flammulina velutipes
Aspergillus proliferans
Aspergillus peyronelii
Aspergillus candidus
Aspergillus janus
Rhizopus stolonifer
Ustilago maydis
Kabatiella zeae
Stagonospora avenae
Septoria tritici
Phyllosticta zeae
Verticillium albo−atrum
Leptosphaerulina trifolii
Thielaviopsis basicola
Pesotum ulmi
Phoma medicaginis
Drechslera tritici−repentis
Phytophthora megasperma
Leptosphaerulina sp.
Umbelopsis versiformis
Stagonospora arenaria
0
1
2
3
4
5
6
µM reducing sugar

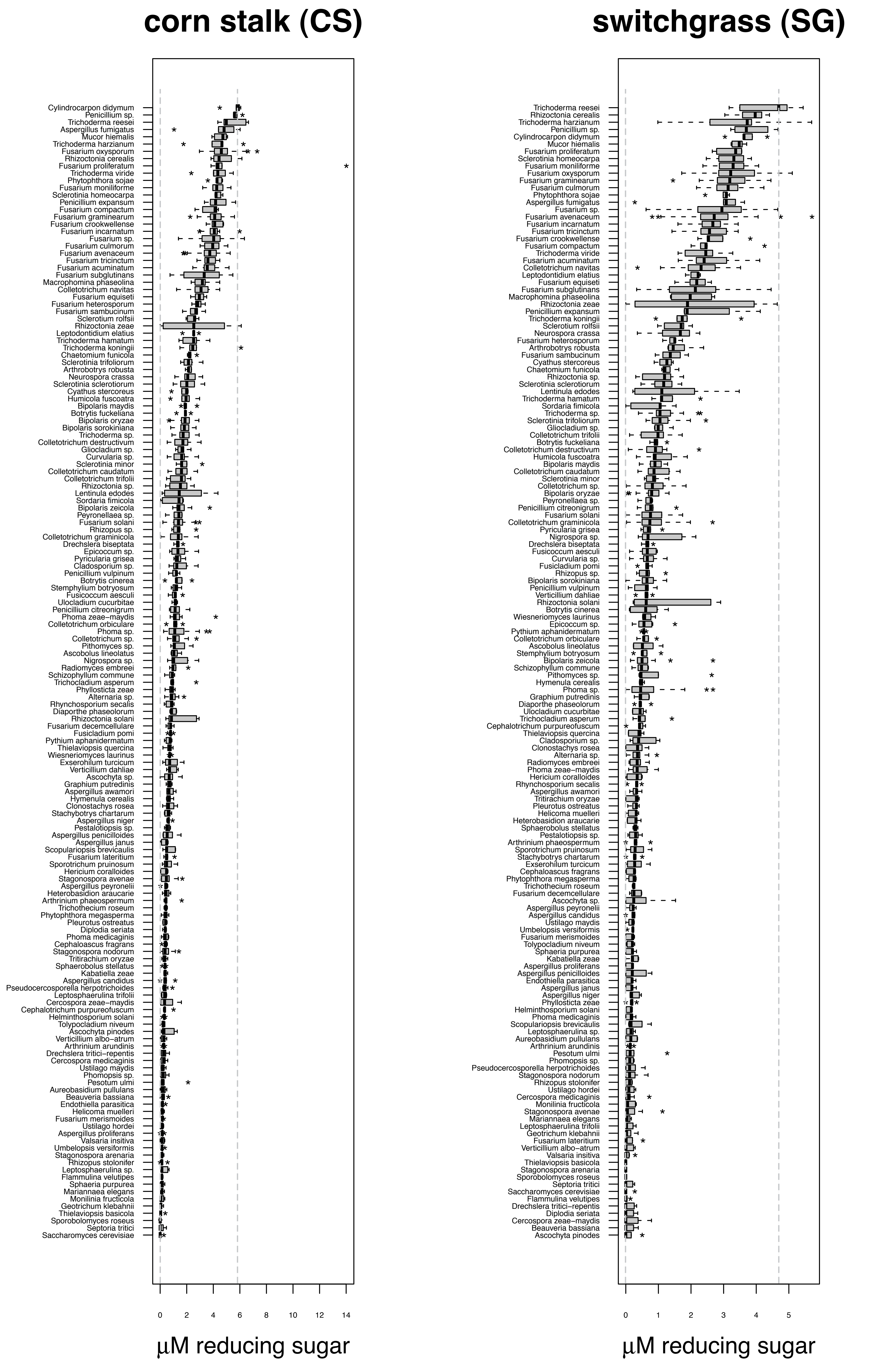
corn stalk (CS)
Cylindrocarpon didymum
Penicillium sp.
Trichoderma reesei
Aspergillus fumigatus
Mucor hiemalis
Trichoderma harzianum
Fusarium oxysporum
Rhizoctonia cerealis
Fusarium proliferatum
Trichoderma viride
Phytophthora sojae
Fusarium moniliforme
Sclerotinia homeocarpa
Penicillium expansum
Fusarium compactum
Fusarium graminearum
Fusarium crookwellense
Fusarium incarnatum
Fusarium sp.
Fusarium culmorum
Fusarium avenaceum
Fusarium tricinctum
Fusarium acuminatum
Fusarium subglutinans
Macrophomina phaseolina
Colletotrichum navitas
Fusarium equiseti
Fusarium heterosporum
Fusarium sambucinum
Sclerotium rolfsii
Rhizoctonia zeae
Leptodontidium elatius
Trichoderma hamatum
Trichoderma koningii
Chaetomium funicola
Sclerotinia trifoliorum
Arthrobotrys robusta
Neurospora crassa
Sclerotinia sclerotiorum
Cyathus stercoreus
Humicola fuscoatra
Bipolaris maydis
Botrytis fuckeliana
Bipolaris oryzae
Bipolaris sorokiniana
Trichoderma sp.
Colletotrichum destructivum
Gliocladium sp.
Curvularia sp.
Sclerotinia minor
Colletotrichum caudatum
Colletotrichum trifolii
Rhizoctonia sp.
Lentinula edodes
Sordaria fimicola
Bipolaris zeicola
Peyronellaea sp.
Fusarium solani
Rhizopus sp.
Colletotrichum graminicola
Drechslera biseptata
Epicoccum sp.
Pyricularia grisea
Cladosporium sp.
Penicillium vulpinum
Botrytis cinerea
Stemphylium botryosum
Fusicoccum aesculi
Ulocladium cucurbitae
Penicillium citreonigrum
Phoma zeae−maydis
Colletotrichum orbiculare
Phoma sp.
Colletotrichum sp.
Pithomyces sp.
Ascobolus lineolatus
Nigrospora sp.
Radiomyces embreei
Schizophyllum commune
Trichocladium asperum
Phyllosticta zeae
Alternaria sp.
Rhynchosporium secalis
Diaporthe phaseolorum
Rhizoctonia solani
Fusarium decemcellulare
Fusicladium pomi
Pythium aphanidermatum
Thielaviopsis quercina
Wiesneriomyces laurinus
Exserohilum turcicum
Verticillium dahliae
Ascochyta sp.
Graphium putredinis
Aspergillus awamori
Hymenula cerealis
Clonostachys rosea
Stachybotrys chartarum
Aspergillus niger
Pestalotiopsis sp.
Aspergillus penicilloides
Aspergillus janus
Scopulariopsis brevicaulis
Fusarium lateritium
Sporotrichum pruinosum
Hericium coralloides
Stagonospora avenae
Aspergillus peyronelii
Heterobasidion araucarie
Arthrinium phaeospermum
Trichothecium roseum
Phytophthora megasperma
Pleurotus ostreatus
Diplodia seriata
Phoma medicaginis
Cephaloascus fragrans
Stagonospora nodorum
Tritirachium oryzae
Sphaerobolus stellatus
Kabatiella zeae
Aspergillus candidus
Pseudocercosporella herpotrichoides
Leptosphaerulina trifolii
Cercospora zeae−maydis
Cephalotrichum purpureofuscum
Helminthosporium solani
Tolypocladium niveum
Ascochyta pinodes
Verticillium albo−atrum
Arthrinium arundinis
Drechslera tritici−repentis
Cercospora medicaginis
Ustilago maydis
Phomopsis sp.
Pesotum ulmi
Aureobasidium pullulans
Beauveria bassiana
Endothiella parasitica
Helicoma muelleri
Fusarium merismoides
Ustilago hordei
Aspergillus proliferans
Valsaria insitiva
Umbelopsis versiformis
Stagonospora arenaria
Rhizopus stolonifer
Leptosphaerulina sp.
Flammulina velutipes
Sphaeria purpurea
Mariannaea elegans
Monilinia fructicola
Geotrichum klebahnii
Thielaviopsis basicola
Sporobolomyces roseus
Septoria tritici
Saccharomyces cerevisiae
0 2 4 6 8 10 12 14
µM reducing sugar
switchgrass (SG)
Trichoderma reesei
Rhizoctonia cerealis
Trichoderma harzianum
Penicillium sp.
Cylindrocarpon didymum
Mucor hiemalis
Fusarium proliferatum
Sclerotinia homeocarpa
Fusarium moniliforme
Fusarium oxysporum
Fusarium graminearum
Fusarium culmorum
Phytophthora sojae
Aspergillus fumigatus
Fusarium sp.
Fusarium avenaceum
Fusarium incarnatum
Fusarium tricinctum
Fusarium crookwellense
Fusarium compactum
Trichoderma viride
Fusarium acuminatum
Colletotrichum navitas
Leptodontidium elatius
Fusarium equiseti
Fusarium subglutinans
Macrophomina phaseolina
Rhizoctonia zeae
Penicillium expansum
Trichoderma koningii
Sclerotium rolfsii
Neurospora crassa
Fusarium heterosporum
Arthrobotrys robusta
Fusarium sambucinum
Cyathus stercoreus
Chaetomium funicola
Rhizoctonia sp.
Sclerotinia sclerotiorum
Lentinula edodes
Trichoderma hamatum
Sordaria fimicola
Trichoderma sp.
Sclerotinia trifoliorum
Gliocladium sp.
Colletotrichum trifolii
Botrytis fuckeliana
Colletotrichum destructivum
Humicola fuscoatra
Bipolaris maydis
Colletotrichum caudatum
Sclerotinia minor
Colletotrichum sp.
Bipolaris oryzae
Peyronellaea sp.
Penicillium citreonigrum
Fusarium solani
Colletotrichum graminicola
Pyricularia grisea
Nigrospora sp.
Drechslera biseptata
Fusicoccum aesculi
Curvularia sp.
Fusicladium pomi
Rhizopus sp.
Bipolaris sorokiniana
Penicillium vulpinum
Verticillium dahliae
Rhizoctonia solani
Botrytis cinerea
Wiesneriomyces laurinus
Epicoccum sp.
Pythium aphanidermatum
Colletotrichum orbiculare
Ascobolus lineolatus
Stemphylium botryosum
Bipolaris zeicola
Schizophyllum commune
Pithomyces sp.
Hymenula cerealis
Phoma sp.
Graphium putredinis
Diaporthe phaseolorum
Ulocladium cucurbitae
Trichocladium asperum
Cephalotrichum purpureofuscum
Thielaviopsis quercina
Cladosporium sp.
Clonostachys rosea
Alternaria sp.
Radiomyces embreei
Phoma zeae−maydis
Hericium coralloides
Rhynchosporium secalis
Aspergillus awamori
Tritirachium oryzae
Pleurotus ostreatus
Helicoma muelleri
Heterobasidion araucarie
Sphaerobolus stellatus
Pestalotiopsis sp.
Arthrinium phaeospermum
Sporotrichum pruinosum
Stachybotrys chartarum
Exserohilum turcicum
Cephaloascus fragrans
Phytophthora megasperma
Trichothecium roseum
Fusarium decemcellulare
Ascochyta sp.
Aspergillus peyronelii
Aspergillus candidus
Ustilago maydis
Umbelopsis versiformis
Fusarium merismoides
Tolypocladium niveum
Sphaeria purpurea
Kabatiella zeae
Aspergillus proliferans
Aspergillus penicilloides
Endothiella parasitica
Aspergillus janus
Aspergillus niger
Phyllosticta zeae
Helminthosporium solani
Phoma medicaginis
Scopulariopsis brevicaulis
Leptosphaerulina sp.
Aureobasidium pullulans
Arthrinium arundinis
Pesotum ulmi
Phomopsis sp.
Pseudocercosporella herpotrichoides
Stagonospora nodorum
Rhizopus stolonifer
Ustilago hordei
Cercospora medicaginis
Monilinia fructicola
Stagonospora avenae
Mariannaea elegans
Leptosphaerulina trifolii
Geotrichum klebahnii
Fusarium lateritium
Verticillium albo−atrum
Valsaria insitiva
Thielaviopsis basicola
Stagonospora arenaria
Sporobolomyces roseus
Septoria tritici
Saccharomyces cerevisiae
Flammulina velutipes
Drechslera tritici−repentis
Diplodia seriata
Cercospora zeae−maydis
Beauveria bassiana
Ascochyta pinodes
0 1 2 3 4 5
µM reducing sugar
