# Supplementary material for: Arsenal of plant cell wall degrading enzymes reflects host preference among plant pathogenic fungi
Source: Biotechnol Biofuels. 2011 Feb 16;4:4. doi: 10.1186/1754-6834-4-4 (PMC3051899; doi:10.1186/1754-6834-4-4)
Supplement: Additional file 4 — Supplemental Figure 3. Hierarchical clustering of complete dataset, excluding Trichoderma reesei. Heatmap showing mean activities and clustering of 155 species of plant pathogenic and non-pathogenic fungi when assayed for hydrolysis of eight polysaccharide substrates [XG, xyloglucan (from tamarind); FP, filter paper; AL, alfalfa; SS, soybean stems; SG, switchgrass; CS, corn stalks; AXW, arabinoxylan (from wheat); XY, xylan (from birch)]. T. reesei RUT-C30 was excluded from this analysis because of its unusual hydrolytic activities. Negative estimations of reducing sugars were adjusted to zero and data were standardized within substrates by subtracting the substrate mean and dividing by the standard deviation. Dendrogram and ordering was determined using the distance matrix computation (dist) and hierarchical clustering (hclust) functions in R. Red colors indicate values greater than the substrate mean, while blue colors indicate values less than the mean. Column Z-score and color intensity indicate how many standard deviations the species mean is from the substrate mean. [file 1754-6834-4-4-S4.PDF]

Figure S3 – Hierarchical clustering of complete dataset, excluding *T. reesei*

Heatmap showing mean activities and clustering of 155 species of plant pathogenic and non-pathogenic fungi when assayed for hydrolysis of eight polysaccharide substrates (XG, xyloglucan (from tamarind); FP, filter paper; AL, alfalfa; SS, soybean stems; SG, switchgrass; CS, corn stalks; AXW, arabinoxylan (from wheat); XY, xylan (from birch)). *T. reesei* RUT-C30 was excluded from this analysis because of its unusual hydrolytic activities. Negative estimations of reducing sugars were adjusted to zero, and data were standardized within substrates by subtracting the substrate mean and dividing by the standard deviation. Dendrogram and ordering was determined using the distance matrix computation (dist) and hierarchical clustering (hclust) functions in R. Red colors indicate values greater than the substrate mean, while blue colors indicate values less than the mean. Column Z-score and color intensity indicate how many standard deviations the species mean is from the substrate mean.

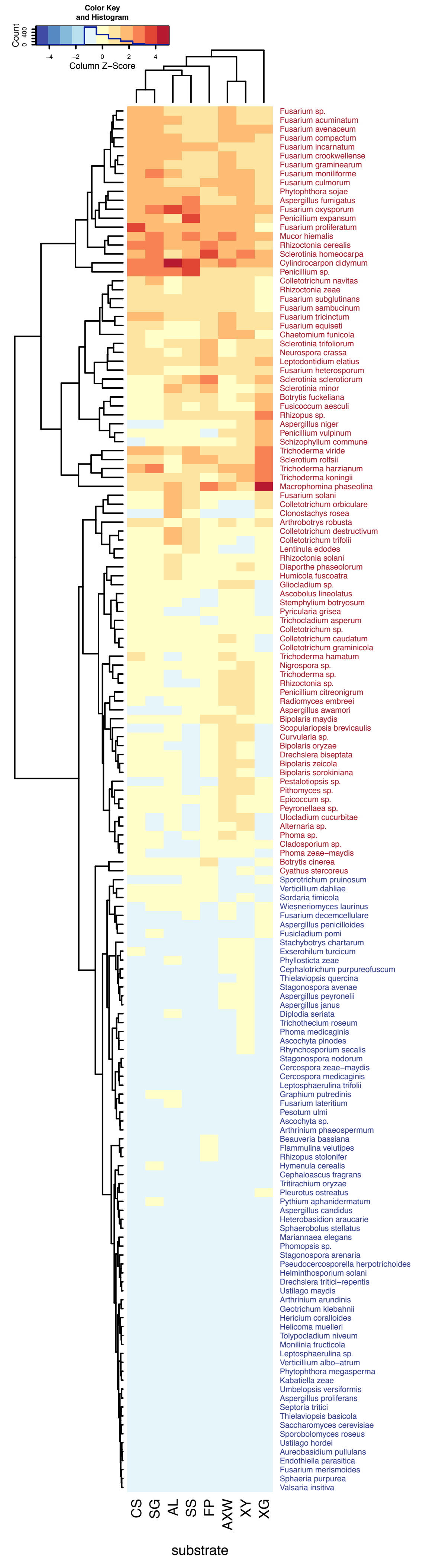
Color Key
and Histogram
Count
0 400
−4 −2 0 2 4
Column Z−Score
Fusarium sp.
Fusarium acuminatum
Fusarium avenaceum
Fusarium compactum
Fusarium incarnatum
Fusarium crookwellense
Fusarium graminearum
Fusarium moniliforme
Fusarium culmorum
Phytophthora sojae
Aspergillus fumigatus
Fusarium oxysporum
Penicillium expansum
Fusarium proliferatum
Mucor hiemalis
Rhizoctonia cerealis
Sclerotinia homeocarpa
Cylindrocarpon didymum
Penicillium sp.
Colletotrichum navitas
Rhizoctonia zeae
Fusarium subglutinans
Fusarium sambucinum
Fusarium tricinctum
Fusarium equiseti
Chaetomium funicola
Sclerotinia trifoliorum
Neurospora crassa
Leptodontidium elatius
Fusarium heterosporum
Sclerotinia sclerotiorum
Sclerotinia minor
Botrytis fuckeliana
Fusicoccum aesculi
Rhizopus sp.
Aspergillus niger
Penicillium vulpinum
Schizophyllum commune
Trichoderma viride
Sclerotium rolfsii
Trichoderma harzianum
Trichoderma koningii
Macrophomina phaseolina
Fusarium solani
Colletotrichum orbiculare
Clonostachys rosea
Arthrobotrys robusta
Colletotrichum destructivum
Colletotrichum trifolii
Lentinula edodes
Rhizoctonia solani
Diaporthe phaseolorum
Humicola fuscoatra
Gliocladium sp.
Ascobolus lineolatus
Stemphylium botryosum
Pyricularia grisea
Trichocladium asperum
Colletotrichum sp.
Colletotrichum caudatum
Colletotrichum graminicola
Trichoderma hamatum
Nigrospora sp.
Trichoderma sp.
Rhizoctonia sp.
Penicillium citreonigrum
Radiomyces embreei
Aspergillus awamori
Bipolaris maydis
Scopulariopsis brevicaulis
Curvularia sp.
Bipolaris oryzae
Drechslera biseptata
Bipolaris zeicola
Bipolaris sorokiniana
Pestalotiopsis sp.
Pithomyces sp.
Epicoccum sp.
Peyronellaea sp.
Ulocladium cucurbitae
Alternaria sp.
Phoma sp.
Cladosporium sp.
Phoma zeae−maydis
Botrytis cinerea
Cyathus stercoreus
Sporotrichum pruinosum
Verticillium dahliae
Sordaria fimicola
Wiesneriomyces laurinus
Fusarium decemcellulare
Aspergillus penicilloides
Fusicladium pomi
Stachybotrys chartarum
Exserohilum turcicum
Phyllosticta zeae
Cephalotrichum purpureofuscum
Thielaviopsis quercina
Stagonospora avenae
Aspergillus peyronelii
Aspergillus janus
Diplodia seriata
Trichothecium roseum
Phoma medicaginis
Ascochyta pinodes
Rhynchosporium secalis
Stagonospora nodorum
Cercospora zeae−maydis
Cercospora medicaginis
Leptosphaerulina trifolii
Graphium putredinis
Fusarium lateritium
Pesotum ulmi
Ascochyta sp.
Arthrinium phaeospermum
Beauveria bassiana
Flammulina velutipes
Rhizopus stolonifer
Hymenula cerealis
Cephaloascus fragrans
Tritirachium oryzae
Pleurotus ostreatus
Pythium aphanidermatum
Aspergillus candidus
Heterobasidion araucarie
Sphaerobolus stellatus
Mariannaea elegans
Phomopsis sp.
Stagonospora arenaria
Pseudocercosporella herpotrichoides
Helminthosporium solani
Drechslera tritici−repentis
Ustilago maydis
Arthrinium arundinis
Geotrichum klebahnii
Hericium coralloides
Helicoma muelleri
Tolypocladium niveum
Monilinia fructicola
Leptosphaerulina sp.
Verticillium albo−atrum
Phytophthora megasperma
Kabatiella zeae
Umbelopsis versiformis
Aspergillus proliferans
Septoria tritici
Thielaviopsis basicola
Saccharomyces cerevisiae
Sporobolomyces roseus
Ustilago hordei
Aureobasidium pullulans
Endothiella parasitica
Fusarium merismoides
Sphaeria purpurea
Valsaria insitiva
CS
SG
AL
SS
FP
AXW
XY
XG
substrate
